# The effect of body weight on the knee joint biomechanics based on subject-specific finite element-musculoskeletal approach

**DOI:** 10.1038/s41598-024-63745-x

**Published:** 2024-06-14

**Authors:** Malek Adouni, Harun Aydelik, Tanvir R. Faisal, Raouf Hajji

**Affiliations:** 1 Biomedical and Instrumentation Engineering, Abdullah Al Salem University, Khalidiya, Kuwait; 2 Physical Medicine and Rehabilitation Department, Northwestern University, 345 East Superior Street, Chicago, IL 60611 USA; 3 Mathematics, College of Integrative Studies, Abdullah Al Salem University, Khalidiya, Kuwait; 4 Department of Mechanical Engineering, University of Louisiana at Lafayette, Lafayette, LA 70508 USA; 5 Internal Medicine Department, Medicine Faculty of Sousse, University of Sousse, Sousse, Tunisia

**Keywords:** Obesity, Gait, Osteoarthritis, Musculoskeletal, Finite element, Biomedical engineering, Computational models

## Abstract

Knee osteoarthritis (OA) and obesity are major public health concerns that are closely intertwined. This intimate relationship was documented by considering obesity as the most significant preventable risk factor associated with knee OA. To date, however, the effects of obesity on the knee joint's passive-active structure and cartilage loading have been inconclusive. Hence, this study investigates the intricate relationship between obesity and knee OA, centering on the biomechanical changes in knee joint active and passive reactions during the stance phase of gait. Using a subject-specific musculoskeletal and finite element approach, muscle forces, ligament stresses, and articular cartilage contact stresses were analyzed among 60 individuals with different body mass indices (BMI) classified under healthy weight, overweight, and obese categories. Our predicted results showed that obesity significantly influenced knee joint mechanical reaction, increasing muscle activations, ligament loading, and articular cartilage contact stresses, particularly during key instances of the gait cycle—first and second peak loading instances. The study underscores the critical role of excessive body weight in exacerbating knee joint stress distribution and cartilage damage. Hence, the insights gained provide a valuable biomechanical perspective on the interaction between body weight and knee joint health, offering a clinical utility in assessing the risks associated with obesity and knee OA.

## Introduction

Osteoarthritis (OA) is a painful disorder affecting diarthrodial joints, resulting from the inadequate and often irregular repair of damaged joint tissue^1^. According to radiographic findings, the prevalence of OA has increased by 25% in Western cultures over the last decade^2–9^. The knee is the most frequently affected joint, with a considerably higher occurrence compared to the hip and ankle joints^10–12^. Given its widespread prevalence, knee OA is poised to be ranked as the fourth leading cause of disability in women and the eighth in men globally^12^. Despite the common occurrence of this condition, the precise causes of knee OA remain under investigation. This complexity primarily arises from the intricate interplay of mechanical and metabolic changes in articular cartilage, bone, and neuromuscular control^13^. Mechanical factors, particularly joint loading during daily activities, have been recognized as pivotal in initiating and advancing OA^14^. Over the last 30 years, significant research has delved into the complex link between OA and walking biomechanics^15–22^. People with knee OA often display biomechanical behaviors that differ markedly from those without the condition^18,23–26^. These include a narrower range of knee flexion, lower maximum knee flexion angles, decreased peak external knee flexion moments, and increased peak knee adduction angles and moments during the walking stance phase. A key aspect connecting obesity with OA involves similar biomechanical effects^27^. Compared to individuals with normal weight, obese people exhibit higher peak knee adduction angles while walking and show less knee flexion in the early stance phase^28–31^. This observation underscores obesity as a primary, modifiable risk factor for OA and expands the traditional link between obesity and knee OA that is limited to the increased compressive forces experienced by the knee joint during weight-bearing activities^32–38^.

As per the latest data extracted from the Behavioral Risk Factor Surveillance System (BRFSS)^39,40^, the global obesity rate has skyrocketed, now standing at ten times the level it was in 1975. Interestingly, the precise impact of obesity on joint biomechanics remains somewhat elusive. Notably, the majority of studies examining the biomechanics of lower extremities in obese individuals have not provided a detailed quantitative analysis of key variables^41–49^. These variables encompass muscle functionality and the distribution of stresses and strains within the joints, both of which could offer valuable insights into the observed shifts in daily activity patterns. To investigate this, these studies have utilized real-time measurements of joint movements combined with three-dimensional link-segment models employing inverse dynamics and optimization techniques. However, it is essential to point out that these studies have not fully accounted for the passive resistance of soft tissues within the knee joint. In other words, these studies have regarded muscles as simplified agents generating forces independently of internal mechanics, and joints have been treated as kinematic constraints that follow the same motion pattern regardless of external loading conditions.

We assert that effective prevention and management of knee OA depend on a thorough grasp of how stress and strain are distributed within the knee's different components, both in normal conditions and when circumstances change, as is the case with overweight and obese individuals. These outcomes are shaped not just by external reactions but also by the actions of the muscles within the joint. Therefore, precision in assessing muscle forces plays a pivotal role in ensuring the accuracy of stress and strain calculations^50,51^. Nevertheless, due to technical challenges in measuring these variables and accounting for the complexities of physiological loads and motions, both in laboratory settings and in live measurements, it remains difficult, particularly when trying to gauge stress and strain in the knee's cartilage and meniscus, as well as the forces acting on its ligaments. Despite prior model studies and in vivo measurements^41–52^, there has yet to be a significant shift in musculoskeletal simulation that effectively combines experimental and computational approaches to fully understand the dynamic relationship between skeletal motion and the internal mechanics of the joint. This understanding is crucial for unraveling the connections between obesity, degenerative joint disease, and OA^53,54^. This framework should deliver vital insights, especially during walking, shedding light not only on muscle forces but also on the distribution of loads and stress and strain patterns within the supportive soft tissues of the joint.

This study's overarching goal is to develop a systematic engineering approach that delves into the simultaneous interplay between body weight categories and the combined mechanical reactions within the knee joint, specifically concerning the load on the articular cartilage. Given that changes in how cartilage bears load are considered predictive of OA developments in the joint, this information becomes crucial for a comprehensive understanding of the factors that potentially contribute to prolonged degeneration and the early onset of OA. We hypothesized that muscle forces, as well as joint passive reactions, will exhibit significant disparities between individuals with unhealthy weights and their healthy-weight counterparts, ultimately resulting in increased joint burden.

## Methods

### Gait data collection and X-ray image acquisition

**Figure 1:**
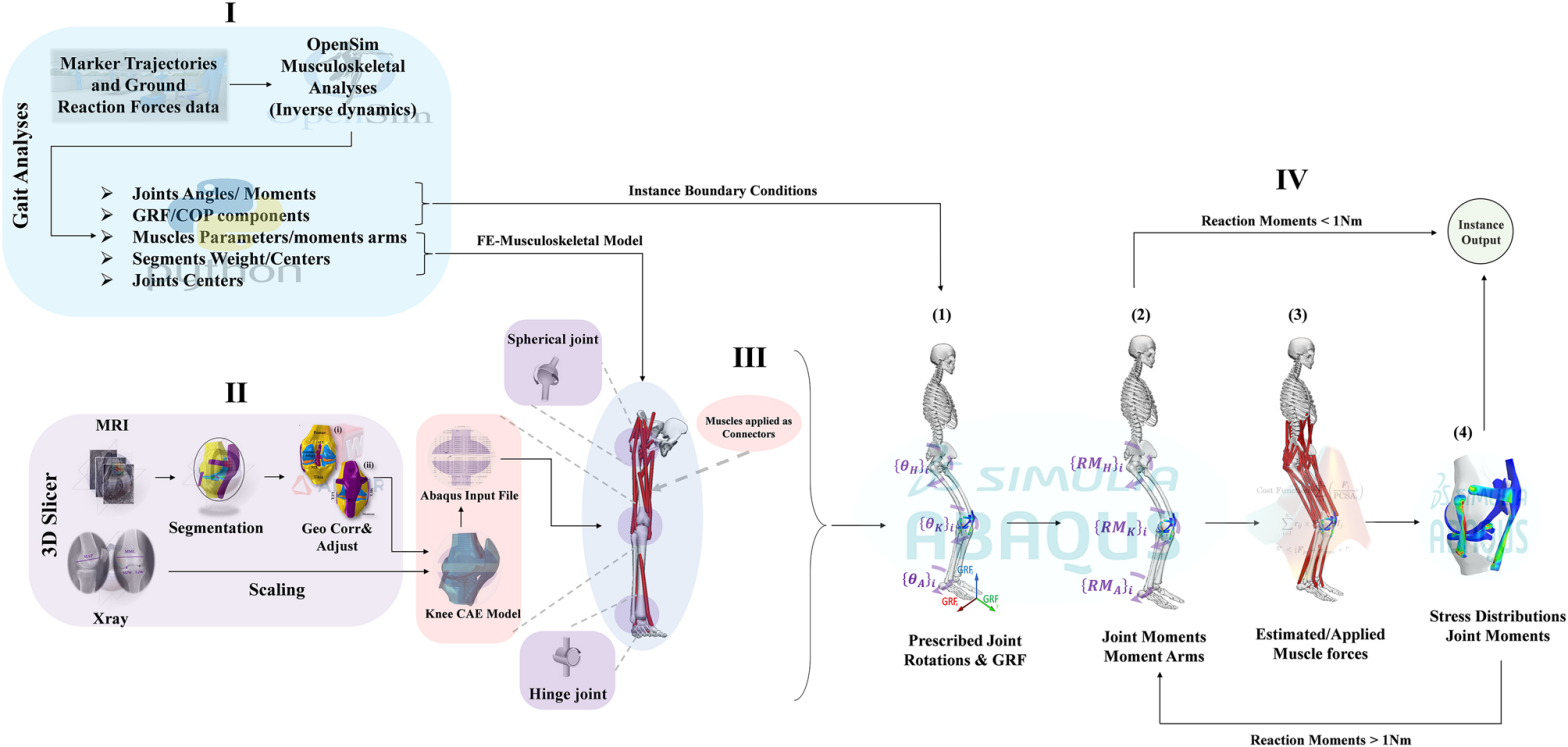
Workflow of the presented study. (I) Lower extremity joint kinematics and kinetics for the healthy weight, overweight, and obese participants (N = 60 participants). (II) Knee model template generation from MRI to geometry correction and adjustments and scaling of the generated template to match anatomical landmarks of the considered subject^65^. (III) FE-musculoskeletal model. (VI) Simulation flow; (1) Prescribed joint rotations and GRF, (2) Computing joint reaction moments and muscle moment arms, (3) Estimated muscle forces and applied as connector tractions^52,108^, (4) Computing joint stress and updated reaction moments. For more information regarding the system of axes, joint center calculations, and muscle characteristics, please refer to^56,109^.

The research study recruited 60 individuals who were matched in terms of age, daily activity levels, and stable body mass (with less than a 2.5 kg change in the previous 3 months). These individuals had never experienced knee pain or undergone lower limb surgery. Prior to conducting the tests, all subjects provided informed consent following the permission (IRC20/21SOEMEPR02) and guidelines of the institutional ethics review board of the Australian University of Kuwait. All experiments were conducted in adherence to relevant guidelines and regulations, following the principles outlined by the Declaration of Helsinki. They were then divided into three groups, each consisting of 20 individuals: healthy weight (BMI below 25), overweight (BMI between 25 and 30), and obese (BMI exceeding 30) (Table 1)^55^. Sagittal and frontal plane X-ray images of the knee joint of each subject were acquired at a fully standing position using a DigitalDiagnost Rel 4.3 (Philips Medical Systems) X-ray device (Fig. 1). Using an optoelectronic motion capture system synchronized with a P6000 force platform (BTS-Bioengineering, Inc.), all participants' external ground reaction forces and three-dimensional motion of the lower limb were measured. Twenty-two spherical reflective markers, each with a diameter of 20 mm, were positioned on specific anatomical landmarks: the shoulder, the greater trochanter, the lateral malleolus, and the lateral epicondyle. Additionally, markers were placed on the foot, shank, thigh, and pelvis segments. These markers, along with virtual markers identified during quiet standing, were used to establish anatomical coordinate systems for each segment of the lower limb^56^. The data collection involved participants walking barefoot at a self-selected speed, with a minimum of five walking trials per participant. For more details, please see the supplementary material section.

**Table 1:** Demographics of healthy weight, overweight, and obese subject groups.

	Healthy weight Mean (SD)	Overweight Mean (SD)	Obese Mean (SD)
Age (years)	32.65 (3.93)	34.22 (5.27)	31.81 (5.34)
Height (m)	1.78 (0.021)	1.76 (0.028)	1.73 (0.025)
Mass (kg)	74.51 (5.29)	85.23 (4.89)	97.62 (5.03)
BMI (kg/m^2^)	21.78 (1.65)	27.57 (1.32)	32.87 (1.88)

### Musculoskeletal model

Each participant's subject-specific musculoskeletal model was generated by scaling a basic model using the dimensions recorded from the lower limb static trial (Fig. 1). This model is characterized by 27 degrees of freedom and consists of 92 muscle–tendon actuators (based on the Gait2392 model in OpenSim). A Hill-type model represented the musculotendon units^57,58^, while the ankle joint was modeled as having 1 degree of freedom, and the hip and knee joints were modeled with 3 degrees of freedom. The OpenSim Python application programming interface (API) and the scaled model were used in each trial to perform inverse kinematic analyses. These analyses involved a least-squares optimization algorithm aimed at minimizing the disparity between the position of each rigid body and the measured marker data^59^ to calculate joint kinematics. The net external moments acting on the lower limb joints were computed using an inverse dynamics approach^56^. The scaled model provided information on joint centers, muscle insertions, moment arms, and maximum isometric forces. The muscle moment arms were identified based on the degrees of freedom of the lower limb joints. All these variables were subsequently utilized in the Musculoskeletal-FE analyses conducted in the study (Fig. 1).

### Musculoskeletal-FE analyses

The subject-specific Musculoskeletal-FE model (Fig. 1) was created by scaling a previously developed and validated model^51,60–63^ using data extracted from the OpenSim model and X-ray measurements. In this model, the hip and ankle joints were represented as three-dimensional and two-dimensional spherical joints, respectively. Surrounding these joints were a total of 31 muscles, with four around the ankle and 27 around the hip^56,64^. On the other hand, the knee joint was modeled using a complex nonlinear approach that incorporated both passive anatomical structures and active components, including eight muscles. This comprehensive model included femoral, tibial, and patellar cartilage, menisci, cruciate ligaments (anterior-ACL and posterior-PCL), collateral ligaments (medial-MCL and lateral-LCL), patellofemoral ligaments (medial-MPL and lateral-LPL), as well as the patellar (PT) and quadriceps (QT) tendons (Fig. 1). To ensure accurate representation, the model was scaled using the offsets mesh-scaling option available in Abaqus. The scaling process took into account the morphological dimensions measured by X-ray participants (Fig. 1), such as the maximum anterior–posterior length of the femoral condyle, maximum tibiofemoral joint space, and maximum medial–lateral distance from the distal femur^65^. Furthermore, the muscle insertion points surrounding the knee joint were adjusted to match those extracted from the OpenSim model, which was utilized in generating the complete Musculoskeletal-FE model. For further details, please refer to the supplementary materials section.

#### Constitutive models

Different constitutive models were utilized to drive the behavior of soft tissues in the study. According to Staubli et al.^66^, it was assumed that the PT and QT had neo-Hookean characteristics with material coefficients of 55.9 MPa and 65.9 MPa, respectively. A transversely-isotropic-hyperelastic material model was employed for the ligaments, assuming almost incompressible behavior^50,63,67^. The model was based on Limbert and Middleton's^68^ ideas of representing the strain energy function without any coupling. In this model, it was assumed that the fibers in the ligaments were extensible, uniformly distributed, and perfectly bonded within the ground matrix. The matrix, on the other hand, was considered to be hyperelastic-isotropic. The strain energy function used in the model excluded any support for compressive loads and incorporated an exponential form to characterize the stiffening behavior of the collagen fibers under tension. Prior investigations^69^ treating the model validation and exploring the sensitivity response of the joint to this material formulation were considered to select the right sets of material parameters in the current study. The model's detailed description was previously demonstrated in other works^69–71^. The ligaments' pre-strains were introduced by decomposing the total deformation gradient into the reference and stress-free states^50^. The menisci were modeled as transversely isotropic, linearly elastic, homogeneous materials^72–74^ with a circumferential modulus of 120 MPa and axial-transverse moduli of 20 MPa. The Poisson's ratios in the circumferential, radial, and axial directions were set to 0.45, 0.3, and 0.3, respectively^50^. Knee articular cartilages were modeled as fibril-reinforced hyperelastic materials, where the properties and orientations of the collagen network varied with depth^75–78^. In the top layer of the cartilage, the collagen fibril networks were arranged in a horizontal mixture, running parallel to the medial and lateral directions. In the transitional zone, the fibril orientations appeared random and gradually shifted from parallel to the surface to perpendicular. In the deep zone, the vertical fibrils were positioned at a right angle to the subchondral junction. For further details on the material formulation, please refer to prior works^61,62,75,79^ and supplementary materials section.

#### Loading and boundary conditions

**Figure 2:**
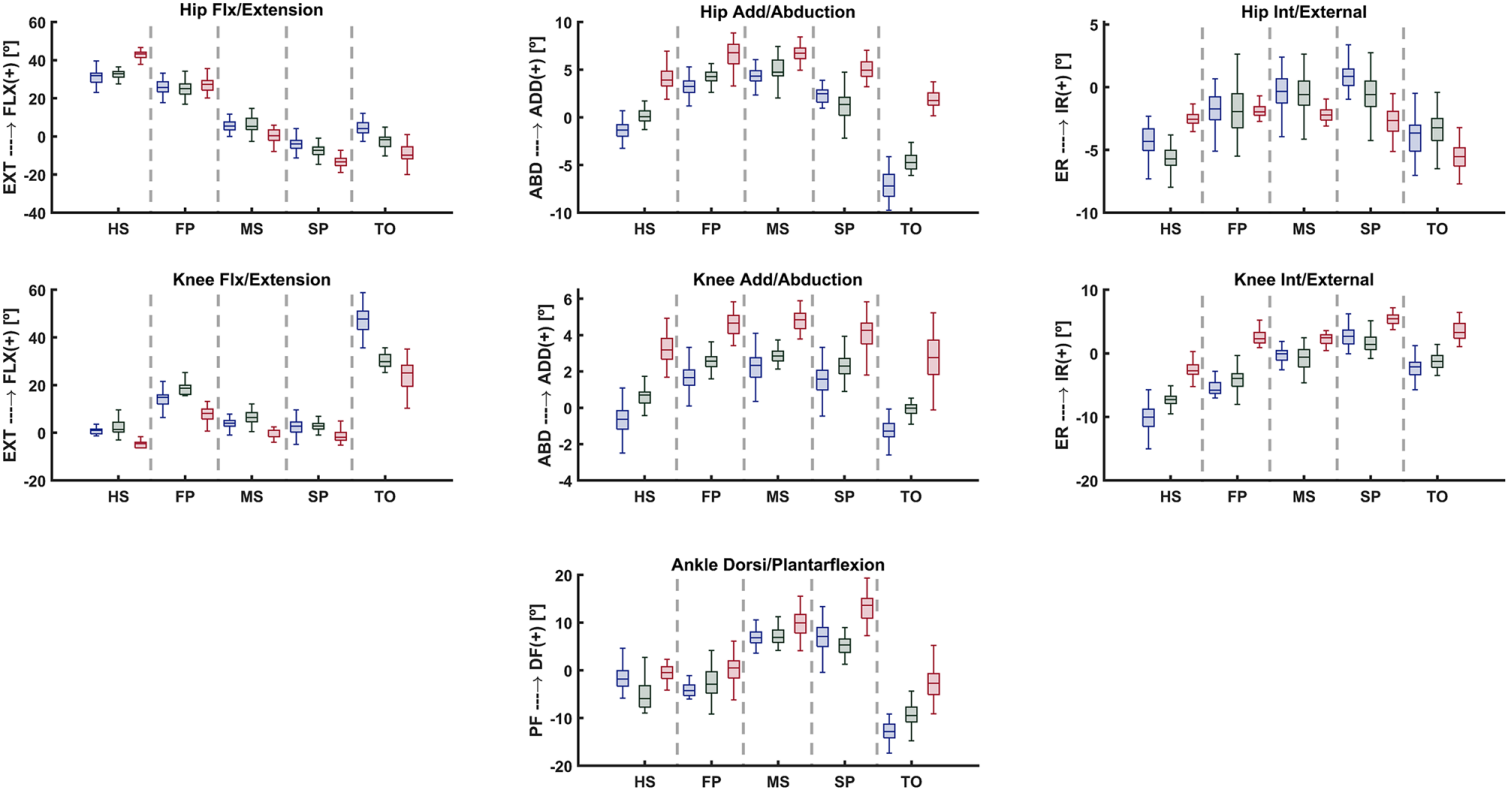
Lower extremity joint rotations for the healthy weight, overweight, and obese participants, in five instances corresponding to the beginning heel strike (HS), first loading peak (FP), midstance (MS), second loading peak (SP), and toe-off (TO) of the stance phase.

**Figure 3:**
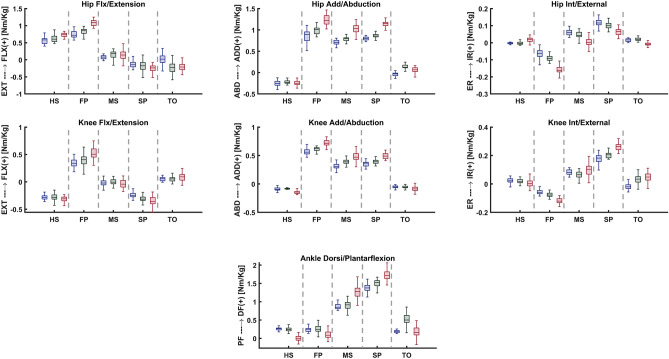
Lower extremity joint moment for the healthy weight, overweight, and obese participants, in five instances corresponding to the beginning heel strike (HS), first loading peak (FP), midstance (MS), second loading peak (SP), and toe-off (TO) of the stance phase.

**Figure 4:**
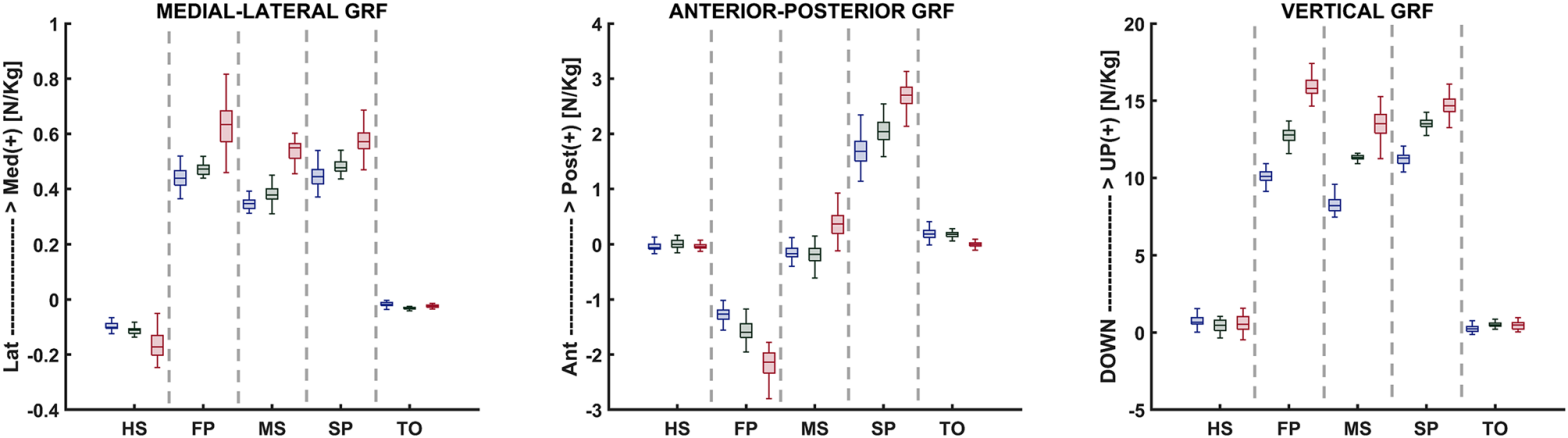
Ground reaction force components for the healthy weight, overweight, and obese participants, in five instances corresponding to the beginning heel strike (HS), first loading peak (FP), midstance (MS), second loading peak (SP), and toe-off (TO) of the stance phase.

The subject-specific Musculoskeletal-FE model employed each participant's ankle/knee/hip joint kinematics and kinetics (rotations/moments) and foot reaction forces to drive the analyses (Fig. 2, 3, 4). Five specific time instances were considered for the analyses, namely heel strike (HS), first loading peak (FP), midstance (MS), second loading peak (SP), and toe-off (TO)^50,52,80^. During each period, the ankle and hip joints, along with the femur, remained constrained in their respective instantaneous positions, while the patella and tibia were unconstrained, except for the prescribed knee joint rotations. The position of the resultant ground reaction force at each moment was adjusted to recreate the external moments acting on the joints. Static optimization was used to predict the muscle forces at the ankle, knee, and hip joints during each stance period. The optimization process incorporated moment equilibrium equations as constraints, considering three equations at the knee and hip joints and one at the ankle joint. The cost function was the sum of cubed muscle stresses of the entire lower extremity^51,81^. This iterative process involved balancing the joint reaction moments in loaded configurations at each instance. After obtaining the muscle forces, they were used as corrective external loads. This process was repeated 6–15 times until convergence was reached, with unbalanced moments not exceeding 0.1 Nm. The analysis employed commercial software tools, specifically Matlab (Optimization Toolbox, genetic algorithms) and ABAQUS 2019 (Static analysis) (Fig. 1). Finally, the study investigated various aspects, including the muscles, ligaments, and contact forces within the knee joint, as well as the average and maximum contact stress and its distribution. For further details, please refer to the supplementary materials section.

#### Statistical analyses

The data were analyzed using the Python NumPy and Scipy data science libraries. A focused bi-comparison approach was adopted to assess differences between control (normal weight), overweight, and obese simulations. Depending on the distribution characteristics confirmed by Shapiro–Wilk tests for normality and Levene's tests for equality of variances, appropriate statistical tests were chosen^82,83^. For normally distributed data, an unpaired t-test was used, while for non-normal data, the Mann–Whitney U Test was applied^71^. These methods were selected to ensure the validity of comparisons while maintaining a predefined significance level of 0.05 to determine significant differences^84,85^.

## Results

**Figure 5:**
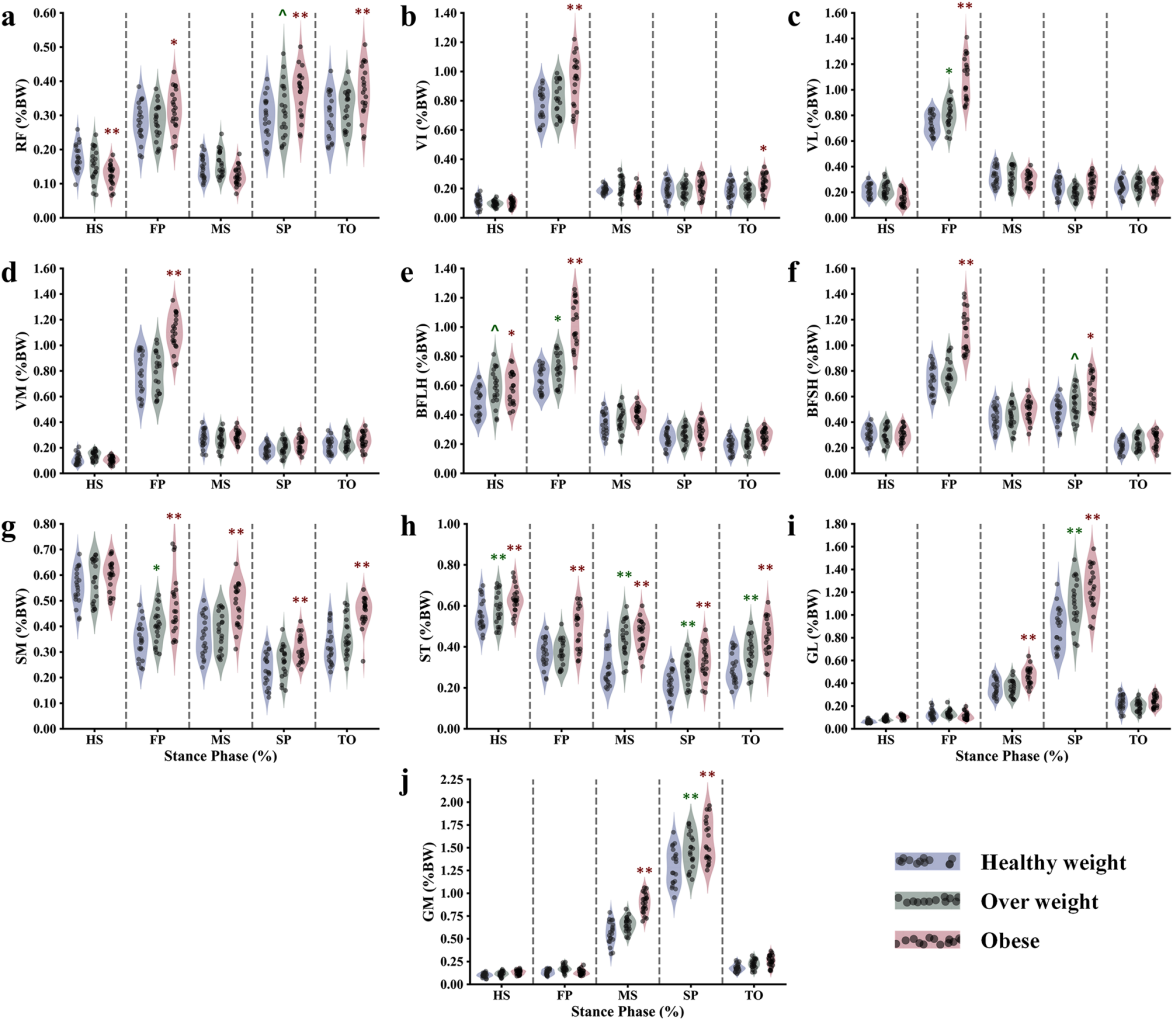
Predicted normalized (/body weight) muscle forces at different periods of stance phase time for the healthy weight, overweight, and obese participants. Quadriceps, RF: rectus femoris (**a**), VI: vastus intermedius (**b**), VM: vastus medialis (**c**), and VL: vastus lateralis (**d**); hamstrings, BFLH: biceps femoris long head (**e**), BFSH: biceps femoris short head (**f**), SM: semimembranosus (**g**), and ST: semitendinosus (**h**); gastrocnemius: MG: medial (**i**) and LG: lateral (**j**). Region statistical significance was indicated for obese vs. healthy weight and overweight vs. healthy weight, **P < 0.001, *P < 0.01, ^P < 0.05.

Figure 5 illustrates the muscle forces observed during the stance phase, highlighting variations among distinct participant groups: the obese cohort, overweight cohort, and healthy control subjects. The data indicate that, apart from the first peak (FP) and toe-off (TO) instances of the stance phase, there are no significant differences in the force generated by the quadriceps' vastus components across all participants (Fig. 5b–d). Notably, the obese cohort exhibits considerably higher forces during the FP instance (p < 0.001). To exemplify, the average force exerted by the vastus lateralis is augmented by 50% in the obese group compared to the healthy control group. Moreover, the peak force in the rectus femoris, occurring later in the stance cycle, is slightly higher in the obese group than in the controls, whereas negligible differences are observed between the overweight and healthy weight groups. In terms of the lateral hamstring, the biceps femoris long head (BFLH) showed significantly higher activation only during the FP instance, with no discernible distinctions among participants during the remaining instances. As for the biceps femoris short head (BFSH), in addition to the FP instance, a noteworthy increase in muscle activity is computed in the second peak (SP) for the obese group (p < 0.01). A gradual level of increase in activity characterizes the medial hamstring components across the three different cohorts, particularly more pronounced in the later stages of the stance cycle. Concerning the gastrocnemius muscles, a significant progressive augmentation is observed only at the midstance and the second peak instance, while no significant differences are noted during the remaining simulated instances (Fig. 5i,j).

**Figure 6:**
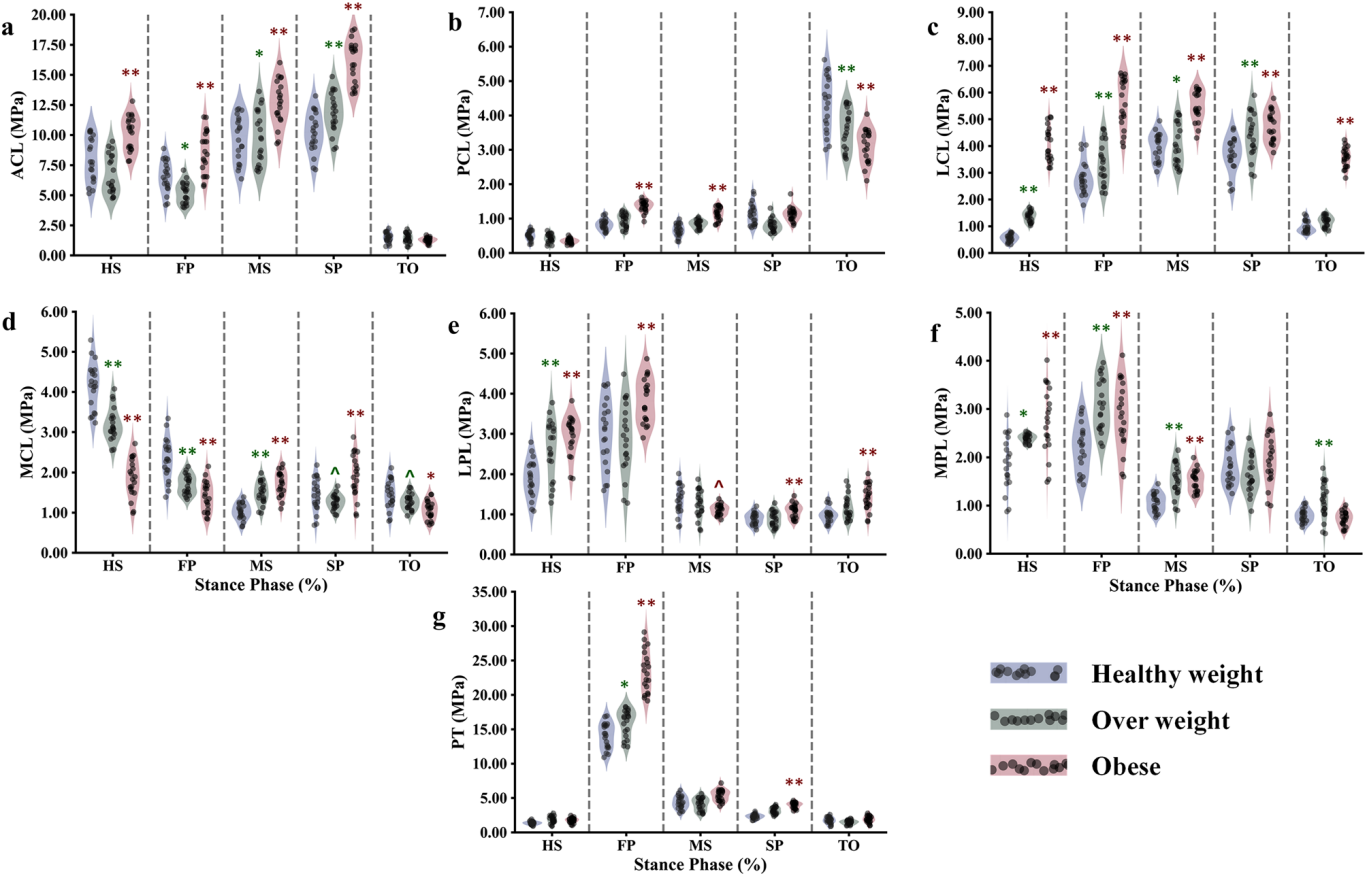
Predicted nominal ligament stresses at different periods of stance phase for the healthy weight, overweight, and obese participants; cruciate ligaments (anterior-ACL (**a**) and posterior-PCL (**b**)), collateral ligaments (Lateral-LCL (**c**) and medial-MCL (**d**)), patellofemoral ligaments (lateral-LPL (**e**) and medial-MPL (**f**)), as well as the patellar tendon (PT (**g**)). Region statistical significance was indicated for obese vs. healthy weight and overweight vs. healthy weight, **P < 0.001, *P < 0.01, ^P < 0.05.

Changes in BMI have a noticeable impact on the nominal stresses experienced by knee ligaments (Fig. 6). Specifically, ACL stresses significantly increase during the stance phase in individuals with obesity, reaching a peak average value of 17.2 MPa at the stance phase's second peak (SP) (Fig. 6a). However, aside from the instances of early and late stance (HS and TO), less pronounced yet still significant differences were observed between individuals who are overweight and those with a healthy BMI. The PCL remains relatively relaxed throughout the stance phase for all participants and only experiences stress at the moment of toe-off (TO), with a progressively significant decrease (p < 0.01) across the three simulated groups. Among the collateral ligaments, the LCL is particularly affected by changes in BMI, exhibiting a significant increase across all simulated instances (p < 0.001). In contrast, the MCL is subjected to much less stress in individuals with obesity compared to those with a healthy BMI, primarily during the early stance phase. The patellofemoral ligaments experience lower stress levels than the tibiofemoral components, and most of the simulated instances show insignificant differences between all participants due to BMI-related changes. A significant increase in stresses on the patellar tendon is observed exclusively in individuals with obesity during the first peak of the stance phase (p < 0.0001). Meanwhile, almost no difference is predicted between individuals with a healthy BMI and those who are overweight (Fig. 6).

**Figure 7:**
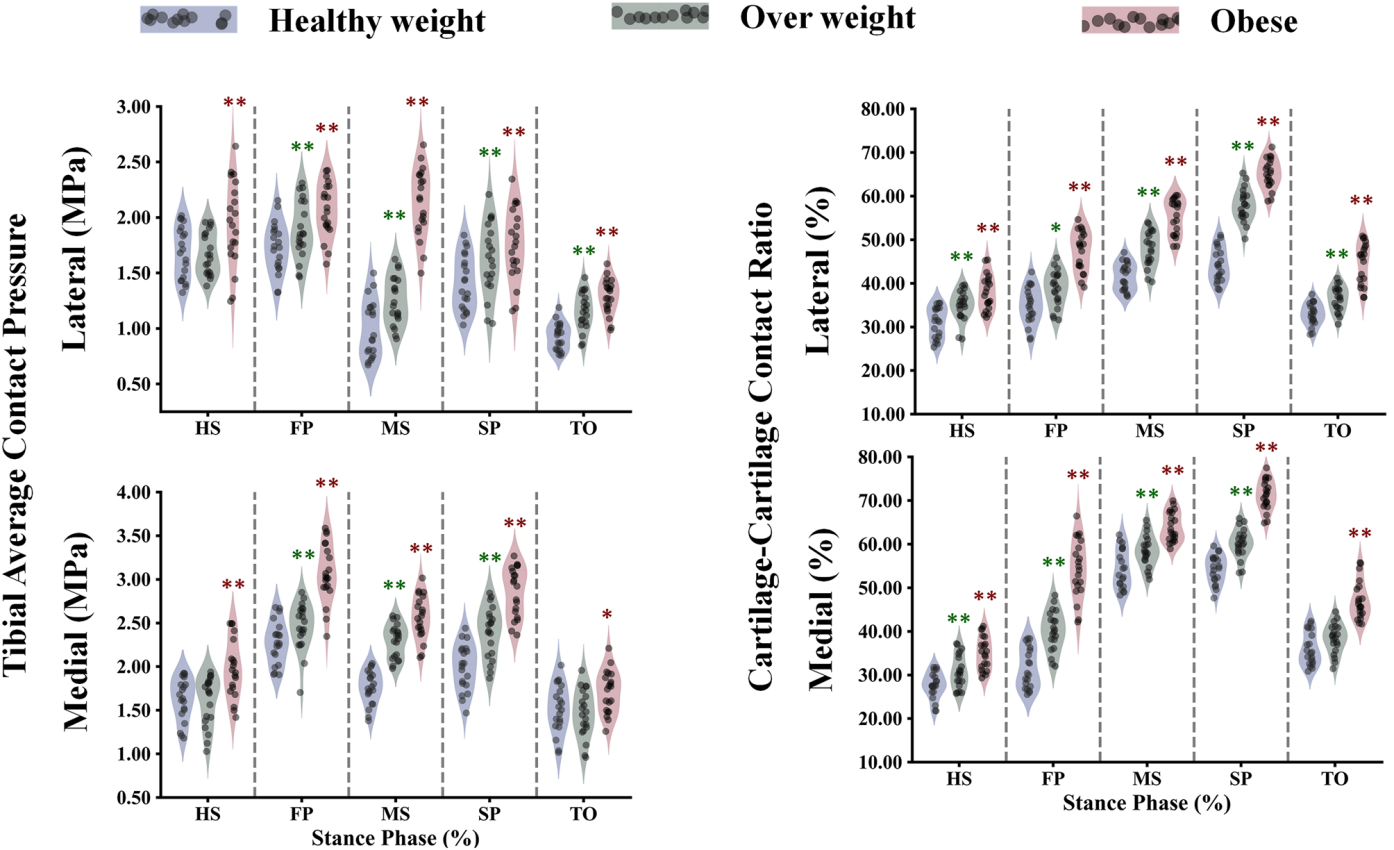
Predicted tibiofemoral (TF) average contact pressure and cartilage–cartilage contact ratio in the medial and lateral plateau at different periods of the stance phase for the healthy weight, overweight, and obese participants. Region statistical significance was indicated for obese vs. healthy weight and overweight vs. healthy weight, **P < 0.001, *P < 0.01, ^P < 0.05.

**Figure 8:**
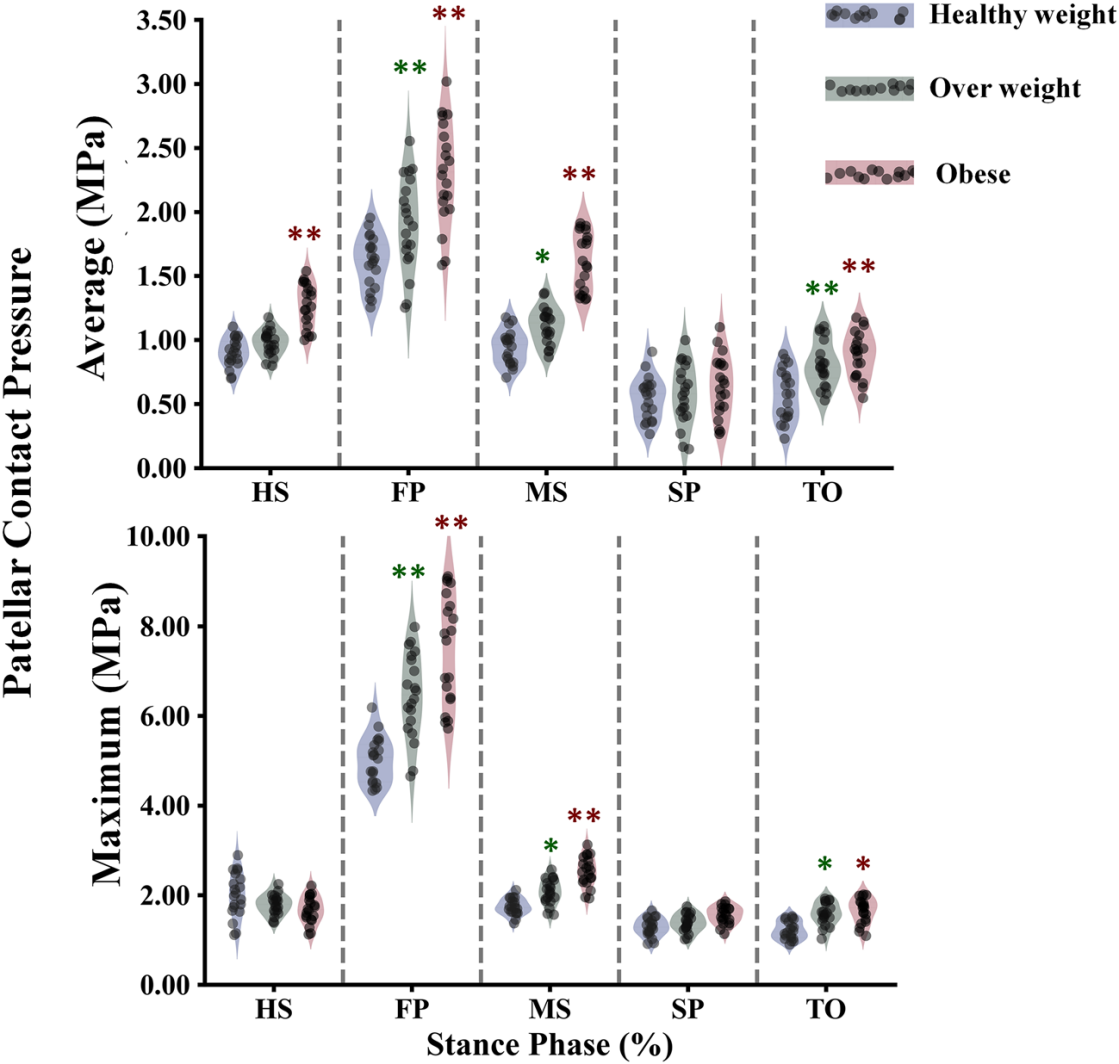
Predicted patellofemoral (PF) average and maximum contact pressure at different periods of stance phase for the healthy weight, overweight, and obese participants. Region statistical significance was indicated for obese vs. healthy weight and overweight vs. healthy weight, **P < 0.001, *P < 0.01, ^P < 0.05.

**Figure 9:**
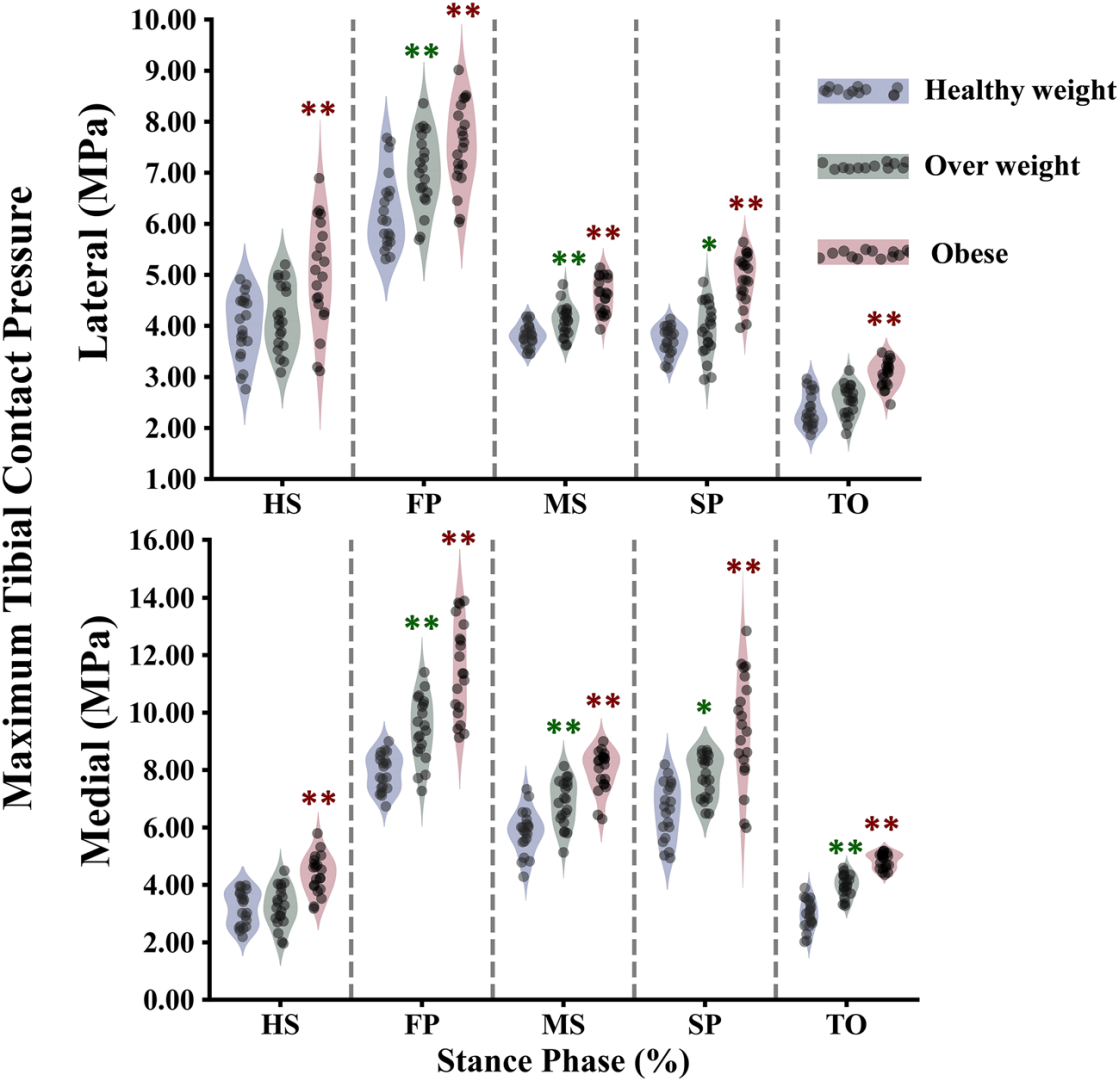
Predicted tibiofemoral (TF) maximum contact pressure in the medial and lateral plateau at different periods of the stance phase for the healthy weight, overweight, and obese participants. Region statistical significance was indicated for obese vs. healthy weight and overweight vs. healthy weight, **P < 0.001, *P < 0.01, ^P < 0.05.

**Figure 10:**
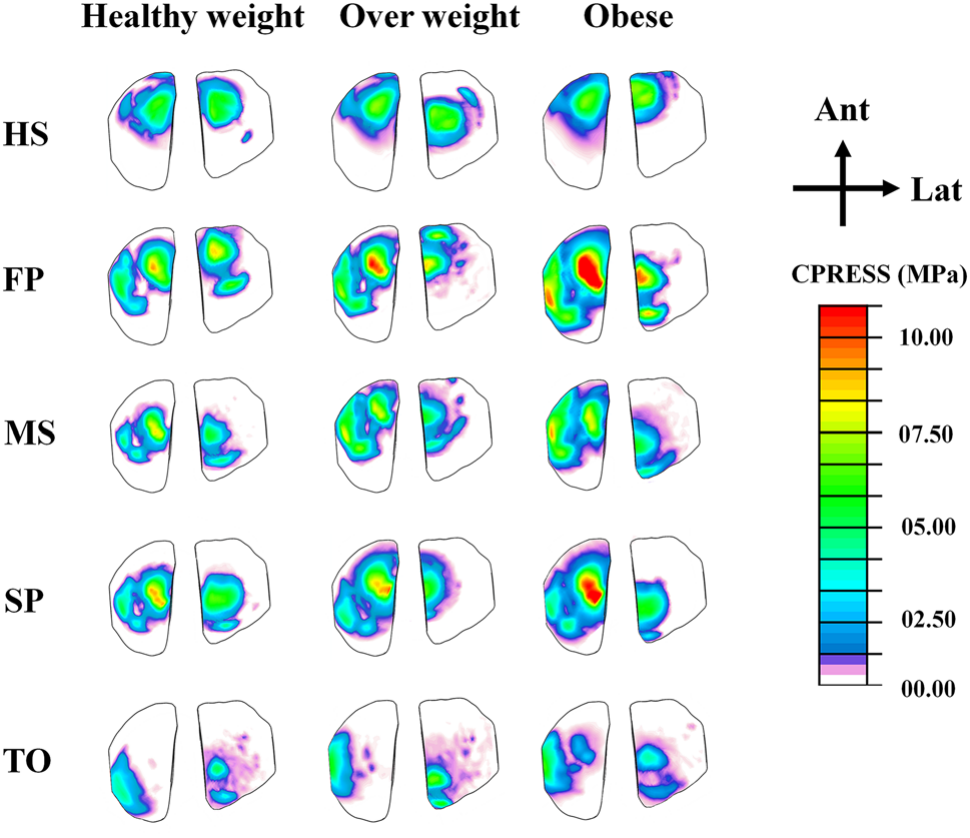
Maximum contact compressive stress at articular surfaces of tibial compartments at different instances for the healthy weight, overweight, and obese participants.

The study calculated all participants' average contact stress in the tibiofemoral and patellofemoral joints (Figs. 7, 8). It was found that obese individuals exhibited a significant increase in stress on the medial compartment during the stance phase, with a maximum value of 3.1 MPa at the FP instance (Fig. 7). On the other hand, the healthy and overweight groups only showed a gradual increase in stress during specific instances (MS and SP). The lateral compartment showed a progressive increase in stress across different groups, except for a notable increase in the obese group at mid-stance. Additionally, in the tibiofemoral joint, the distribution of stress on the non-dumped side (cartilage-cartilage contact) was higher among obese subjects compared to healthy individuals between the HS and TO instances (Fig. 7). The patellofemoral joint had lower average stress compared to the tibiofemoral joint in all simulated cases, with a more significant progressive increase among different groups during the first half of the stance phase (Fig. 8). Furthermore, the tibiofemoral joint experienced much higher maximum contact stresses compared to the patellofemoral joint (Figs. 8, 9). In the FP and SP instances, the medial compartment exhibited greater stress, particularly in obese subjects, with maximum values of 14 MPa and 12 MPa, respectively. The lateral side showed a more gradual increase in stress when comparing different groups. Throughout the stance phase, the stress distribution in the tibiofemoral joint followed a consistent pattern, shifting from the anterior to posterior and lateral to the medial side (Fig. 10).

## Discussion

While there is clear evidence demonstrating a heightened risk of knee joint disorders, specifically cartilage degeneration and progressive OA, among overweight and obese individuals, the underlying mechanisms driving the development and progression of these disorders remain poorly elucidated. Previous research indicates that overweight and obese individuals undergo distinct changes in their walking patterns and resultant joint forces compared to those with a healthy weight^43,86^. However, the precise impact of these altered gait patterns, including stride characteristics, joint angles, and net moments, on the distribution of passive loading within and between the soft tissues of the knee joint during walking remains uncertain. Understanding how these soft tissue loads vary with increasing BMI categories is crucial for investigating the potential role of obesity in the pathways leading to the development and progression of joint diseases. In the current study, prediction confirms the hypothesis that muscle forces, as well as joint passive reactions, exhibited significant disparities between individuals with unhealthy weights and their healthy-weight counterparts, ultimately resulting in increased joint burden.

The present study showed that the estimated forces produced by the three primary muscle groups surrounding the knee joint tended to be higher with increasing BMI category, although the differences were not statistically significant except during peak loading moments (Fig. 5). For the FP instance, which was driven by the combined activation of the quadriceps and hamstring components, significant augmentation of the forces generated by the vastus muscles (Intermedius, lateralis, and medialis) was computed. The vastus lateralis muscle showed a much greater increase in activity in most simulated obese subjects here because this muscle plays an important role in balancing the additional knee flexion and adduction moments^87^. A similar trend was predicted on the antagonist side of the quadriceps, the biceps femoris components. Here, it is interesting to note that the poly-articular role of these muscles in calibrating the internal hip flexion and knee adduction moment additionally generated by obese individuals may explain the predicted higher activation of the biceps femoris. The second half of the stance phase was characterized by a new reorganization of muscle activities, where the gastrocnemius took control of the joint stability. The computed forces of this group of muscles were set on mainly at the mid-stance and reached maximum contribution in the second peak instance. In contrast to the observed jump in the vastus and the biceps femoris activation at the FP instance, a more progressive shape of activation was predicted with gastrocnemius at the SP instance. This soft augmentation was due to the similarly computed slope in the knee extension and ankle dorsiflexion moment between different participants. Also, similar soft augmentation of the biceps femoris short head component was computed at the SP instance, explained by the knee extension and the slight agonist activation of the rectus femoris^53^. Almost insignificant or slight augmentation was computed for the rest of the muscles and simulated instances (Fig. 5).

In general, our study showed a jump in the quadriceps and hamstring muscle activations in early stance with obese participants, while less and more progressive augmentation was computed with most muscles in late stance. The study findings suggest that during the initial stance phase, there is an observed increase in muscle forces to enhance joint stability when a single limb accepts the additional weight associated with excess body mass. Furthermore, as the stance phase progresses, higher forces are generated by the gastrocnemius muscle, potentially indicating a greater need for propulsive force due to the increased body mass. In both the early and late stance phases, these elevated muscle activities may serve as a compensatory strategy adopted by obese individuals, suggesting a potential lack of confidence in joint stability and utilization when compared to individuals who are healthy or even overweight^88,89^. However, the computed augmentation of the muscle forces over the stance phase is still less than the expected one if it is compared with the percentage of the achieved mass augmentation (average of 25% for overweight and 45% for obese participants), which is endorsing earlier observation where some obese individuals reorganizing their neuromuscular function to most likely reduce the muscles fatigue possibility and joint loading^34,90^. Our predicted results are consistent with previous EMG measurements^91^. However, some other investigations reported even lower muscle activation in obese individuals^92,93^. This differential observation may be linked to the recruited subjects, where some evidence reported that silent OA symptoms in obese individuals are responsible for less muscle activation during gait^18^. In addition to that, followed experimental protocols, inconsistency in the obesity history may generate muscle fatigue, and errors in EMG measurements may contribute to the reported differences.

**Figure 11:**
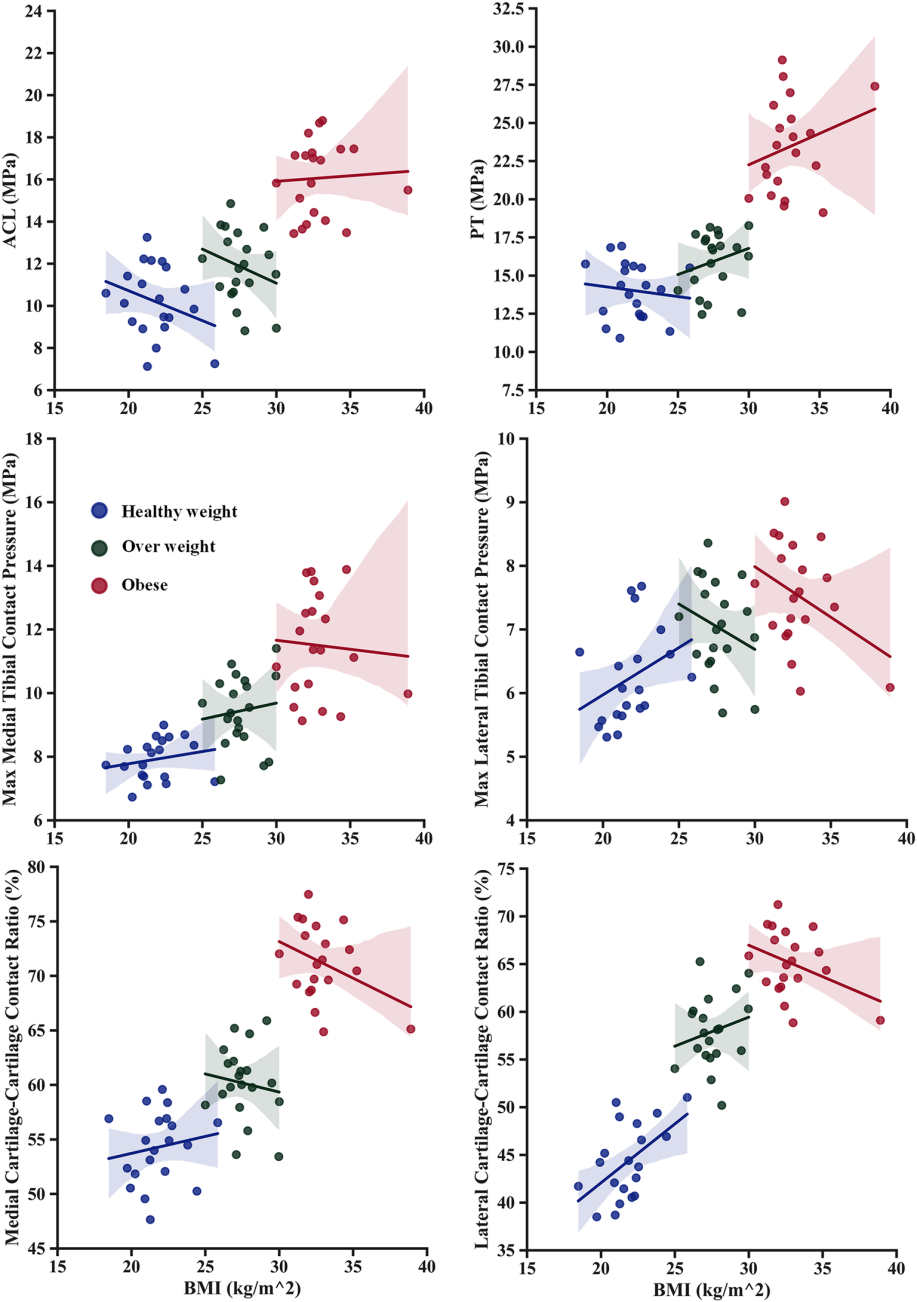
Regression analyses for BMI onto ACL, PT nominal stress, medial and lateral maximum contact pressure, medial and lateral cartilage–cartilage contact ratio.

The computed loads on the knee cruciate ligaments (ACL and PCL) followed nearly opposite trends during the stance phase of gait. The ACL supported the majority of the load from the early stance to the second peak of the cycle, while the PCL remained slack throughout the cycle, only activating at the transition to the swing phase (Fig. 6). This predicted behavior reflects the muscle activation surrounding the knee joint during the stance phase. The mutual activation of the quadriceps and gastrocnemius muscles represents a good explanation of the ACL continued load over the stance phase, where these two were considered antagonist activators of the ACL^94^. The ACL load significantly increased in the obese category (Fig. 11), while the PCL decreased. The higher ACL nominal stresses were linked to the observed augmentation of the muscle forces and decreased flexion angles of the knee joint with obese participants. Collateral ligaments also followed a mutual loading pattern, where the MCL loaded at the early stance only and the LCL over the whole cycle except the HS instance. The MCL nominal stress decreases gradually between the considered BMI categories. However, a clear jump of the same stress was computed in the LCL with obese participants. The substantial augmentation of the knee angular adduction in obese individuals may explain the higher sustained load supported by the LCL over the stance cycle^15^. Patellar tendon nominal stress followed the same trend as the forces of the quadriceps, where the maximum values were computed at the FP instance. Significantly higher stresses were computed with obese participants in this instance (Fig. 11), reflecting the higher activation of the vastus components. Patellofemoral ligaments were much less stressed than the rest in all simulated cases and less differentiated within different considered BMI categories. The two first maximum values of computed ligament stress, 19 MPa for the ACL and 30 MPa for the PT, during this investigation, were near 50% of the elastic limit in both cases, given that the additional burden on the ligament by the obesity not representing a critical loading scenario for these tissues^95^.

The kinematics, kinetics, and neuromuscular adaptation followed by the obese individual during walking significantly affected the contact mechanism in the TF and PF joints (Figs. 7, 8). Because the quadriceps and hamstring muscles are seen as one of the main contributors to the knee joint contact loading during early stance, average compressive stresses were significantly higher with obese participants compared with healthy control in both TF compartments and PF interactions. A more progressive augmentation of the average compressive stresses was computed except for the mid-stance and first peak instances in the lateral and medial plateaus. In these latter, a clear jump in the average stresses was computed with obese individuals, while almost insignificant change was observed between overweight and healthy groups. During the stance cycle, the PF average stress progressively increased with BMI changes, reaching its maximum value at the FP instance. This behavior correlates positively with what was computed in the quadriceps and PT loading. Similar patterns of maximum contact pressure were estimated for all simulated cases in the TF and PF joints. These maximum stresses were much higher with obese subjects (Fig. 11), mainly in the first and second peak instances, reaching a maximum value of 15 MPa on the medial plateau, which is still far from the critical stress the cartilage may support^96^. This result supports earlier hypotheses that higher joint loading due to massive mass augmentation would not be required to initiate cartilage damage in a healthy joint, whereas it may be sufficient to further progress an already diseased joint^97^. On the side of TF load distribution, the medial TF plateau supports most load during the stance cycle in all simulated BMI categories. This is a normal reaction to the frontal angular behavior of the knee joint during the stance phase of gait, which is characterized by more adduction rotation. The kinematics of the obese individual favor a clear increase in adduction rotation, leading to a higher mechanical response on the medial plateau compared to healthy controls. Moreover, mechanical tissue responses at femur-to-tibia contact regions (cartilage-cartilage contact) were significantly greater than those at the meniscus-to-tibia contact regions, mainly after the acceptance phase during stance. This proportion of cartilage-to-cartilage contact ratio keeps increasing progressively with increased body mass (Figs. 7, 11). Both observations represent a good explanation of the reported close link between OA prevalence and obesity^98^. The higher prevalence of cartilage defects in the contact regions between the femur and tibia, as opposed to the contact regions between the meniscus and tibia, diagnosed in obese subjects^98^, may be attributed to this phenomenon.

This study has several limitations that merit discussion. The average BMI of our obese group was 33.6 kg/m^2^, which is lower than the BMI range typically examined in previous studies on obesity^12,29,34^. Consequently, our results may only reflect individuals within this specific BMI range, which could explain the discrepancies observed between our findings and those of prior studies. The prior power analysis was conducted using published data that focused on differences between healthy and obese subjects, who had characteristics slightly different from those of the participants in this study. These differences may affect the predicted results^99–102^. Additionally, the number of subjects was constrained by available funding and laboratory capacity. We also acknowledge the omission of a gait reliability test, which could impact the consistency and accuracy of our gait analysis results^103^. It is important to consider that the use of skin-mounted markers introduces potential errors in determining segment position and orientation due to skin movement artifacts. This limitation applies to all studies employing this methodology, particularly when studying obese participants^104^. The model utilized in this study is based on certain assumptions that are difficult to validate. The material parameters of the knee's soft tissue can vary among patients and at different joint sites, which can influence the magnitude of local tissue mechanical responses. Unfortunately, there are currently no practical methods available to fully extract the mechanical properties of the subject's soft tissue. Therefore, we adopted material parameters from existing literature. Additionally, we used a single geometrical template rather than subject-specific templates based on previous evidence of its effectiveness^65^. Finally, it is important to note that the developed model only considers transient responses, as it does not account for the biphasic behavior of the meniscus and articular cartilage.

In conclusion, the results of this study represent accumulating evidence that underscores the significant link between higher body mass index (BMI) and the exacerbation of knee OA. Here, obesity-related alterations in joint kinematics-kinetics during gait manifest in heightened activation of the lower extremity musculature, increased ligament loading, and a significant augmentation in mean and peak articular cartilage contact stresses. Also, the predicted knee joint stress distribution catalyzes cartilage damage propagation. Consequently, the established biomechanical framework and the inspected parameters in this study are pivotal, offering a clinical utility in assessing the individual-specific impact of weight and weight gain on cartilage reaction and in discerning the potential risks for the onset and advancement of knee OA. In addition, the current investigation not only delineates the intricate biomechanical interplay between obesity and knee OA during the gait cycle but also highlights the advanced knee modeling and simulation pipeline we utilized. This Musculoskeletal-FE analysis, demonstrated for both healthy and unhealthy weight subjects, opens the door for the development of unprecedented diagnostic precision, enabling more targeted interventions^54,105^. If it is well developed, clinicians and therapists can leverage this tool to design personalized rehabilitation protocols and activity modifications, tailoring load conditions to optimize joint health based on individual tissue mechanics^106^. Furthermore, this approach empowers healthcare providers to implement customized educational and preventive care strategies, enhancing patient understanding and compliance^107^. The potential for extending further research and development in these analyses will underscore its value, paving the way for innovative treatments and preventive measures that could transform knee OA management, making it more proactive and effective^106^.

### Supplementary Information

Supplementary Information.

## Data Availability

The submitted manuscript includes all the essential data. Should you require further information, please feel free to contact the corresponding author (malek.adouni@northwes-tern.edu), who will ensure you receive the necessary details.
