# Supplementary material for: The effect of body weight on the knee joint biomechanics based on subject-specific finite element-musculoskeletal approach
Source: Sci Rep. 2024 Jun 14;14:13777. doi: 10.1038/s41598-024-63745-x (PMC11178890; doi:10.1038/s41598-024-63745-x)
Supplement: Supplementary file 1 — Supplementary Information. [file 41598_2024_63745_MOESM1_ESM.pdf]

Supplementary Materials

1. Gait Analysis.

1.1 Study Design and Setting

This research utilized a quantitative, comparative approach to examine active and passive knee joint mechanical reactions among various BMI categories. The research and all measurements took place in Kuwait from 2020 to 2022 at the Australian University Scientific Research Centre's biomechanics and biomodelling unit in partnership with the Physical Medicine WHospital.

1.2 Power analysis

Using data ranges previously reported in the literature for outcomes treating the impact of obesity on the biomechanics of the lower extremities during gait [1-5], a preliminary power analysis was performed using G*Power [6, 7] which gave a conservative estimate varying from 6 to 18 subjects in each group to be sufficient to detect meaningful differences between groups with a power of $1 – \beta = 0.80$. This estimate is consistent with sample sizes reported for similar studies [8, 9].

1.3 Eligibility Criteria

1.3.1 Inclusion Criteria

The criteria for participant inclusion were as follows: only males between the ages of 18 and 40 years were eligible. To qualify, participants must have maintained a stable body weight, with a variation of less than 2.5 kg in the last three months. They also need to engage in less than 30 minutes of moderate physical activity each week and not more than three days per week. Additionally, candidates with no previous history of knee pain or lower limb surgeries were considered. Finally, only those who gave informed consent were included in the study.

1.3.2 Exclusion Criteria

Exclusion criteria for the study were as follows: participants who had a history of knee pain or had undergone any lower limb surgeries were not eligible. Also, those who participated in moderate physical activity for over 30 minutes on at least three days per week were excluded. Individuals whose body weight had shifted by more than 2.5 kg within the previous three months were also ruled out. Additionally, participants displaying signs of osteoarthritis in their knee joints, as indicated by X-ray images, were excluded from the study.

1.4 Subject population

Based on the power analysis, inclusion, and exclusion criteria outlined previously, the research recruited 60 male participants who were similar in age, daily activity levels, and body mass stability (as shown in Table 1 of the manuscripts). The participants were recruited via announcements and advertisements at the university. Before the tests were conducted, all subjects provided informed consent in accordance with the guidelines set by the institutional ethics review board. The study participants were organized into three groups of 20, sorted by BMI according to WHO standards (referenced in Table 1 of the manuscript) [10]. The first group included individuals with a BMI under 25, identified as being within a healthy weight range. The second group, classified as overweight, consisted of participants with a BMI ranging from 25 to 30. The third group, defined as obese, included individuals with a BMI over 30. For the imaging process, all participants stood fully upright as X-ray images of their knee joints were taken in both the frontal and sagittal views using the DigitalDiagnost Rel 4.3 from Philips Medical Systems [11]. A rheumatologist and an orthopedist thoroughly reviewed the X-ray images to verify that there was no apparent connection between osteoarthritis and obesity in any of the participants.

1.5 Data Collection

In this research, the external ground reaction forces and 3D movements of the lower limbs were measured (between 9 am and 1 pm) using the P6000 synchronized force platform and an optoelectronic motion capture system, both provided by BTS-Bioengineering, Inc. The motion capture equipment included eight SMART-DX EVO cameras, which were recording at a frequency of 100 Hz. We placed twenty-two spherical reflective markers, 20 mm in diameter, on vital anatomical landmarks, including the acromion, ASIS, sacrum, greater trochanter, femoral condyle, fibula head, lateral malleolus, fifth metatarsal head, and heel. Additional bar markers were affixed to the thigh and shank segments [12-14]. These markers, along with virtual markers established during periods of quiet standing, were employed to create anatomical coordinate systems for each lower limb segment [15, 16]. Participants walked barefoot at a self-chosen speed, completing at least five trials to ensure adequate data collection. The cutoff frequencies for the ground reaction forces and marker coordinates were 15 Hz and 6 Hz, respectively [17].

2. Musculoskeletal model

An iterative kinematics-driven lower limb musculoskeletal model that accounts for the active-passive structures of the knee joint was developed. The hip and ankle were modeled as three-dimensional and two-dimensional spherical joints, respectively, surrounded by 31 muscles (4 around the ankle and 27 around the hip) [15, 18]. At the same time, the knee joint was represented by a complex nonlinear model consisting of full anatomical passive and active (8 muscles) structures (Sfig. 1).

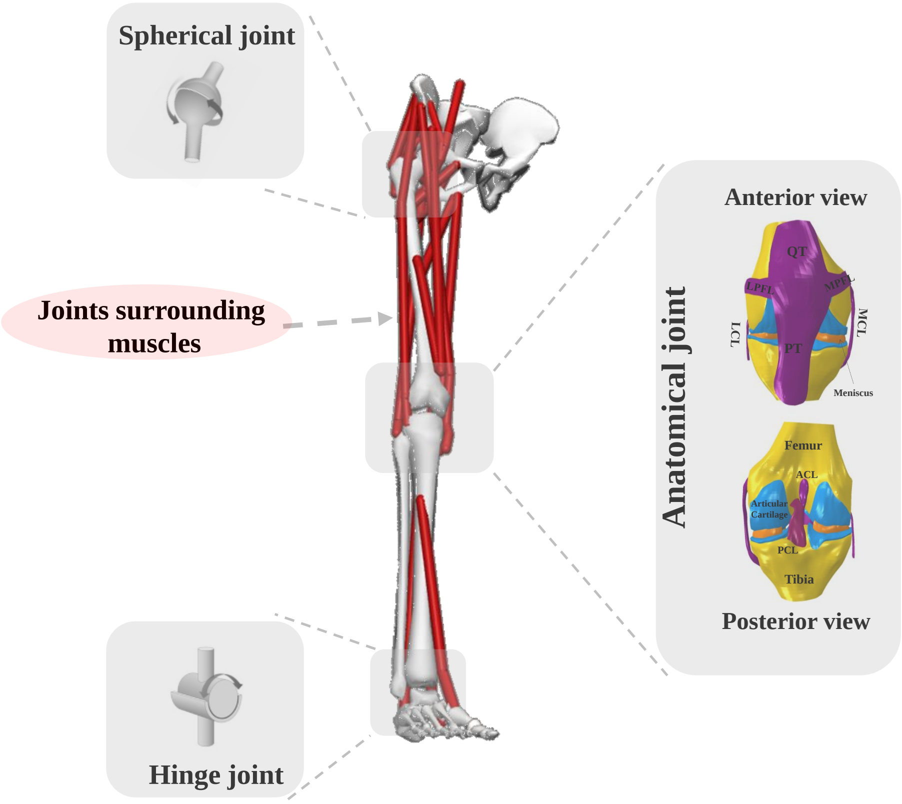

Supplementary Figure S(1): Lower extremity musculoskeletal model, including the hip and ankle as spherical and hinge joints with an anterior and posterior view of the knee's three-dimensional finite element model.

3. Knee Model:

3.1 Geometry and meshing:

An anatomically accurate model of knee joint consisting of the femur, tibial and patellar bones, articular surfaces, as well as the origins and insertions of the ligaments, was derived from digitized magnetic resonance image (MRI) transverse contours (OpenKnee public domain repository at Simtk.org). The knee specimen (female subject: Age 70 years; Height =170 cm;

Weight =77 kg) was scanned at Cleveland Clinic (Biomechanics laboratory) using a one Tesla extremity MRI scanner (Orthone, ONI Medical Systems Inc, Wilmington MA). A scanning protocol was used to provide a good contrast for the soft tissues within the same scan. The protocol characteristics are presented in Supplementary Table S(1).

Supplementary Table S(1): The magnetic resonance imaging settings (OpenKnee).

**Prescan Parameters**	**Sagittal**	**Axial**	**Coronal**
Prescan	Auto	Auto	Auto
Center freq.	Peak	Peak	Peak
**Scan Parameters**	**Sagittal**	**Axial**	**Coronal**
Pulse Sequence	GE3D	GE3D	GE3D
FoV	150	150	150
BW	20	20	20
Frequency	260	260	260
Phase	192	192	192
TR	30	30	30
TE	8.9	8.9	8.9
Flip Angle	35	35	35
Time	5.03	3.19	3.30
Echo Train	1	1	1
NEX	1	1	1
**Scan Options**	**Sagittal**	**Axial**	**Coronal**
Graphics SL	Y	Y	Y
Minimum T.E.	Y	Y	Y
No phase wrap	Y	Y	Y
R.F. spoiling	Y	Y	Y
Flow comp	N	N	N
Magnetic transfer	N	N	N
Partial data	N	N	N
Inversion recovery	N	N	N
Spatial saturation	N	N	N
Fat Suppression	N	N	N
**Slice Parameters**	**Sagittal**	**Axial**	**Coronal**
Number of slices	70	45	60
Slice thickness (mm)	1.5	1.5	1.5
Gap (mm)	0	0	0
Range (mm)	105	67.5	90

The knee was placed in full extension, and the scanning process employed a 3D spoiled gradient-echo sequence with fat suppression, T.R. = 30, T.E. = 6.7, Flip Angle = 200, Field of View (FOV) = 150 mm X 150 mm, Slice Thickness = 1.5 mm. The imaging was conducted in three anatomical planes: (axial, sagittal, and coronal). About 18 minutes was spent finishing the process of scanning. These images are optimal to differentiate between the musculature, tendons, tissue fascia, and bone [19]. The image data set was then imported into an MRI viewing and segmentation analysis package (3D slicer 4.8) and re-sampled in the sagittal, coronal, and axial planes. The muscle-bone junctions were identified from the MRI images following the procedure

outlined in Dhaher and Kahn [20]. Polygonal surfaces were used to generate a F.E. mesh of the knee joint using the Hypermesh (Altair Engineering, Troy, MI) and SOLIDWORKS (CAD) pre-processor. The structure of the tibiofemoral joint was adjusted to match the given dimension in the open knee public domain repository at Simtk.org [19]. Bones were defined as rigid bodies [21] using 4-node quadrilateral elements that injunction with elastic boundaries with the articular cartilages. Eight-node hexahedral elements were used to represent the articular cartilages, ligaments, and menisci (Sfig. 2, 3).

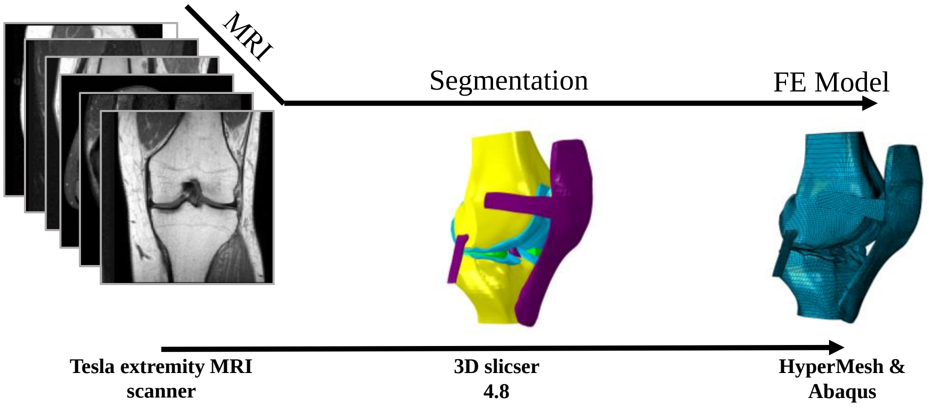

Supplementary Figure S(2): The workflow of building the 3D F.E. model of the knee joint; Clinical magnetic resonance image (MRI) of a knee joint, segmentation process, CAD file, and finite element model (F.E.)

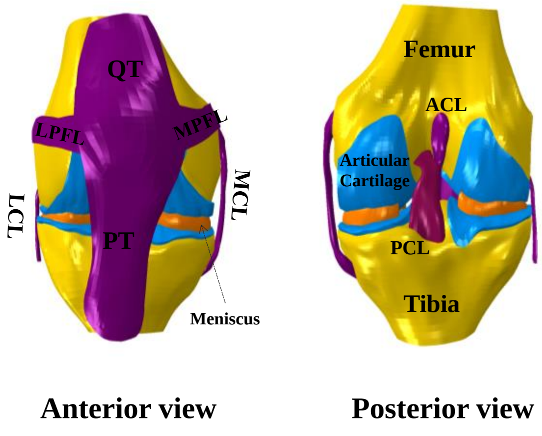

Supplementary Figure S(3): Anterior and posterior views of the three-dimensional finite element model of the knee showing the corresponding soft tissues and articular surfaces acting on the bones. Anterior cruciate ligaments (ACL), posterior cruciate ligaments (PCL), medial and lateral collateral ligament (MCL, LCL), lateral patellofemoral (LPFL), medial patellofemoral

(MPFL), quadriceps tendon (Q.T.), and patellar tendon (P.T.) cartilage layers and menisci are shown. More details on the system of axes and the joint center calculations can be found in [19].

The mesh of the model was obtained through a sensitivity analysis, where a maximum of 6% difference in the von-Mises stress was considered (STable. 2).

Supplementary Table S(2): Mesh of the knee joint.

Set	Number of elements	Types of elements
**Femoral cartilage**	34452	C3D8R
**Tibial cartilage**	17694	C3D8R
**Patellar cartilage**	8736	C3D8R
**Meniscus**	11440	C3D8R
**ACL**	32768	C3D8R
**PCL**	41984	C3D8R
**LCL**	53248	C3D8R
**MCL**	40960	C3D8R
**MPL**	3165	C3D8R
**LPL**	2795	C3D8R
**PT**	32800	C3D8R
**QT**	5117	C3D8R
**Bones (femur, tibia and Patella)**	27560	S4R

3.2 Fibril orientation

For each C3D8R element, a local coordinate system was used to define fibril orientation and implemented by a Python script. This script read the connectivity of each element and defined the local cross-sectional plane (x', y') and its normal vector along the local z'-direction (Sfig. 4).

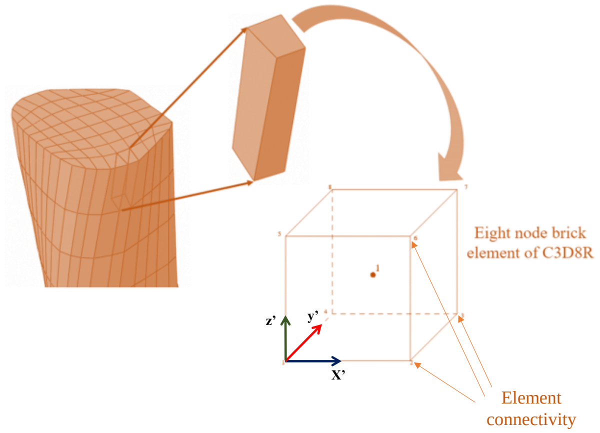

Supplementary Figure S(4): The element local coordinate system (x', y', z') employed to define the orientation of the fibril.

3.3 Knee Model scaling

To match the representation of the subject's knee structure, the finite element (F.E.) knee model underwent morphing to align with the subject's anatomical measurements, as illustrated in Supplementary Figure S(5). This morphing process utilized anisotropic scaling, specifically by multiplying the nodal coordinates (x, y, z) by a percentage difference ratio. This ratio was calculated using the subject's measured dimensions, namely the maximum anterior-posterior (MAP) length, maximum medial-lateral (MML) length, and the maximum tibiofemoral joint space width (MJW=max [MJW, LJW]), represented as (Dx, Dy, and Dz), in relation to the original model's dimensions [22]. To execute the scaling, the mesh editing tool in Abaqus software was employed, coupled with a custom Python subroutine, enabling iterative adjustments to the model.

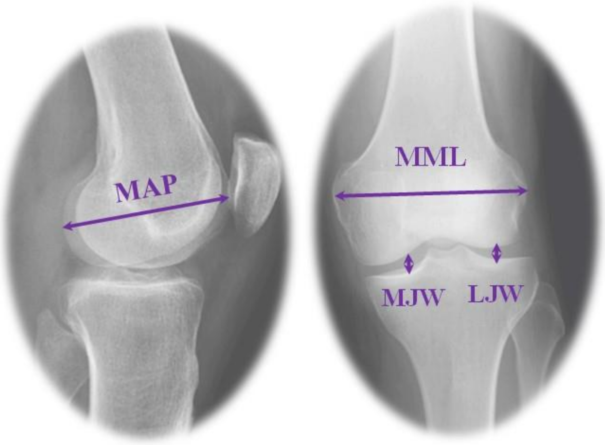

Supplementary Figure S(5): measurements of the anatomical dimensions from the X-ray.

3.4 Interaction and loading analyses.

The explicit algorithm was used during all the simulations with a short step time (0.01s) to mimic quasi-static analysis. The boundary conditions were applied gradually using an exponential function for the normalized amplitude (Sfig. 6) to attain smooth results. Frictionless interaction property was considered for driving surface-to-surface contact formulation. Computations were performed using an Intel(R) Core (T.M.) I9-12900KF@CPU3.20GHz, 64.0 GB of RAM.

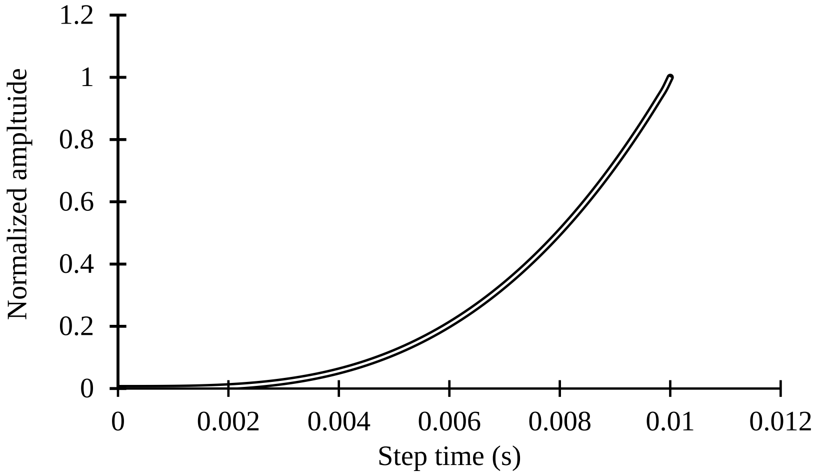

Supplementary Figure S(6): The normalized amplitude as a function of the step time used to guide the application of boundary conditions during knee modeling.

4. Constitutive Models of the Soft Tissue

4.1 Cartilage

The articular cartilage was modeled using incompressible hyperelastic fibrils reinforced composites behavior described by Sajjadinia et al., [23]. The Cauchy stress (σ^c) in the used model was decomposed into a non-fibrillar (σ^{nf}) and fibrillar (σ_i^f) parts as follow:

$$\begin{cases} \sigma^c = v_f \sigma^f + (1 - v_t)\sigma^{nf} & \\ \sigma^{nf} = \eta_0^s \left[-\frac{lnJ}{6J} G_m \left(\frac{3\eta_0^s \, lnJ}{\eta_0^s - 1} - 3\frac{J+\eta_0^s}{J-\eta_0^s} - 1 \right) I + \frac{G_m}{J}\left(FF^T - J^{2/3} I\right) \right] + \frac{1}{D}(J-1)^2 & \\ \sigma_i^f = \left\{ \frac{\eta_0^s}{J} \, ln\varepsilon_f \left(E_0 \varepsilon_f + E_\varepsilon {\varepsilon_f}^2 \right)(n \otimes n) \right\}_i & {\varepsilon_f}_i \succ 0 \\ \sigma_i^f = 0 & {\varepsilon_f}_i \preccurlyeq 0 \end{cases} \quad (1)$$

Where F and J are the deformation gradient tensor and the volumetric deformation, respectively. n and ε_f are the current direction and logarithmic strain of the fibril, respectively. E_0 and E_ε are the collagen stiffening coefficients (initial and strain-dependent). G_m is the shear

modulus and η_0^s is an elastic material constant, v_f is the relative collagen fibril volume fraction. The collagen networks were defined as primary and secondary bundles of fibrils based on their orientation relative to the articular cartilage depth (Sfig. 7). The fibrils were oriented perpendicular to the subchondral junction and turned gradually in the middle zone to become parallel to the articular surface. For more details on the formulation of the material, please see prior works [23, 24]. A list of the properties of the material is presented in Supplementary Table S(3).

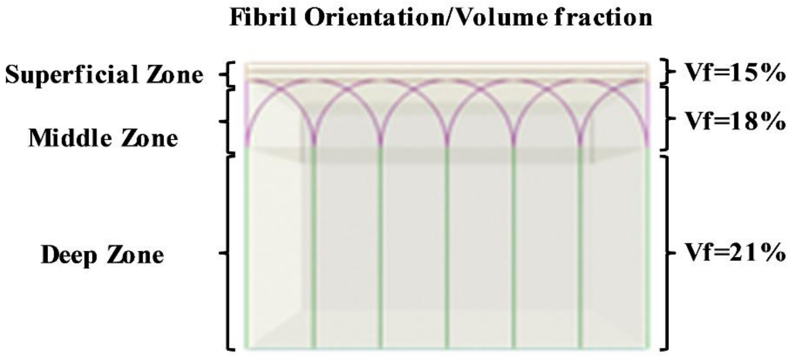

Supplementary Figure S(7): Diagram showing the orientation and volume fraction of the collagen fibrils as a function of the depth in the articular cartilage.

Supplementary Table S(3): Articular cartilage materials properties.

Material parameters	
$\boldsymbol{E_0(MPa)}$: Initial collagen coefficients	4.63
$\boldsymbol{E_\varepsilon(MPa)}$: Strain-dep collagen coefficients	3670
$\boldsymbol{G_m(MPa)}$: Shear modulus	0.723
$\boldsymbol{v_f}$: Collagen fibril volume fraction[1]	$v_t \frac{3}{13}$ or $v_t \frac{1}{13}$
$\boldsymbol{v_t}$: Total depth-dependent collagen volume fraction[2]	$1.4z^2 - 1.1z + 0.59$
$\boldsymbol{\eta_0^s}$: Elastic material constant	$0.1z + 0.1$
$\boldsymbol{D}$: Incompressibility penalty parameter	0.0001

[1] $\frac{3}{13}$ for the primary fibril and $\frac{1}{13}$ for the secondary one.
[2]z: Normalized Depth of the articular cartilage (starting from the cartilage-bone junction area)

4.2 Meniscus

Since the isotropy of the transverse and axial plans in the meniscus has been thoroughly described, a particular subclass of orthotropy, transverse isotropy, was employed to represent the mechanical behavior of this substance [25-27]. The local system axis of the meniscus is defined by axial, transverse, and circumferential axes, with the assumption that the transverse-axial plane is isotropic. As a result of this assumption, the number of independent constants in the matrix

equals 5. To accomplish this, the transverse isotropy requires circumferential modulus (E_C), transverse and axial modulus (E_t=E_a), Poisson's ratio (ν_{ct}= ν_{ca}), which is defined as the ratio of the contractile strain in the transverse plane to the tensile strain in the circumferential direction under the load in the circumferential direction; Poisson's ratio ν_{ta}, which is the Poisson's ratio within the transverse plane and shear modulus G (STable. 4 & Sfig. 8). The stress-strain representation is defined as follows:

$$\begin{Bmatrix} \varepsilon_{11} \\ \varepsilon_{22} \\ \varepsilon_{33} \\ \gamma_{12} \\ \gamma_{13} \\ \gamma_{23} \end{Bmatrix} = \begin{bmatrix} 1/Et & -\vartheta\mathrm{ct/Et} & -\vartheta\mathrm{ta/Ec} & 0 & 0 & 0 \\ -\vartheta\mathrm{ct/Et} & 1/Et & -\vartheta\mathrm{ta/Ec} & 0 & 0 & 0 \\ -\vartheta\mathrm{ta/Et} & -\vartheta\mathrm{ta/Et} & 1/Ec & 0 & 0 & 0 \\ 0 & 0 & 0 & 1/Gt & 0 & 0 \\ 0 & 0 & 0 & 0 & 1/Gc & 0 \\ 0 & 0 & 0 & 0 & 0 & 1/Gc \end{bmatrix} \begin{Bmatrix} \sigma_{11} \\ \sigma_{22} \\ \sigma_{33} \\ \sigma_{12} \\ \sigma_{13} \\ \sigma_{23} \end{Bmatrix} \quad (2)$$

A list of the properties of the material is presented in supplementary Table S(4).

Supplementary Table S(4): Meniscus materials properties

**E_c (MPa)**	**E_t (MPa)**	$\boldsymbol{\nu_{ct}}$	$\boldsymbol{\nu_{ta}}$	**G_t (MPa)**
120	20	0.3	0.2	47

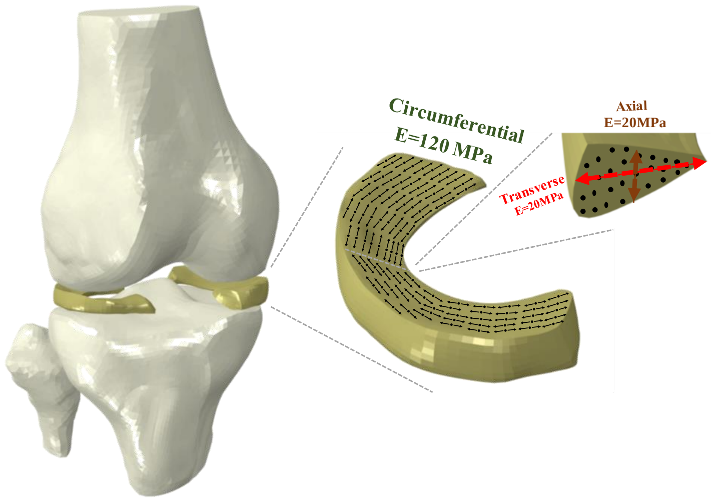

Supplementary Figure S(8): Representation of the circumferential, transversal and axial directions within the meniscus structure [28].

4.3 Ligaments

The knee tendons (PT and QT) were assumed to be neo-Hookean, with material coefficients (C_{10}) of 55.9 MPa for the PT and 65.9 MPa for the QT [29]. Meanwhile, knee ligaments were modeled using an incompressible transversely isotropic hyperelastic behavior [30] via an uncoupled representation of the strain energy function [31]. The collagen fibers were uniformly distributed and properly bound to the isotropic and hyperelastic ground substance. The suggested strain energy function provides a silent reaction under any compressive loads and nonlinear stiffening behavior under tension as follows:

$$\begin{cases} \psi_t(\bar{I}_1,\bar{I}_4,\bar{J}) = \psi_{nf}(\bar{I}_1) + \psi_f(\bar{I}_4) \\ \psi_{nf}(\bar{I}_1,\bar{J}) = c_1(\bar{I}_1 - 3) + \frac{1}{D}(\bar{J} - 1)^2 \\ \psi_f(\bar{I}_4) = \frac{c_2}{2c_3} \exp\left(c_3(\bar{I}_4 - 1)^2\right) \quad if\ \bar{I}_4 > 1 \\ \bar{I}_4 = \bar{F}^T\bar{F} : (n_0 \otimes n_0) \end{cases} \tag{3}$$

Where ψ_{nf}, ψ_f and ψ_{vol} are the strain energy's non-fibrilar, fibrillar, and volumetric parts. n_0 is the fiber orientation in the reference configuration, F is the deformation gradient tensor, c_1, c_2, c_3 are the materials coefficients, and D is the incompressibility penalty parameter. The pre-strains behavior was incorporated into the ligaments by decomposing the deformation gradient ($F = F_0 F_r$) into a stress-free state (F_0) and reference state (F_r). The pre-strains here were defined as the initial stretch (α_0) field with $F_0 = diago\left[\alpha_0 \;\; \sqrt[-1]{\alpha_0} \;\; \sqrt[-1]{\alpha_0}\right]$ [28]. The current study considered the sets of material parameters from our recent publication [28].

5. Knee Model Validation

A tibial axial compression (2000 N), pure adduction (6 deg), and internal (12 deg) rotations at zero extension and joint passive flexion (0-90 deg) were simulated to test the validity of the developed 3D knee model in predicting joint passive response under isolated full joint loading condition. All femur degrees of freedom and only the tibial flexion-extension were fixed during these analyses. In addition, these boundary conditions were fixed to facilitate the comparison with the few experimental data reported in the literature [32-51].

The predicted load-deflection curve of the tibial shaft exhibits a nonlinear stiffening behavior, as shown in Supplementary Figure S(9a). The computed axial deflection has a relatively

higher increase at the initial equilibrium position under ligaments prestress and low axial load, then tend to stabilize at a higher load. On the side of the tibial plateaus load distribution, a higher load has been computed on the lateral compartment (~60%) than the medial one (~40%). The lateral plateau's covered area (meniscus cartilage) was the main supporter of the load, while on the medial plateau, almost the same proportions were computed between the covered and uncovered (cartilage-cartilage) areas. The total knee contact area increased from 404 to 1283 mm^2 when the tibial axial load was augmented by 2000 N (Sfig. 9b). Average contact pressure on the tibial plateau reached its maximum value of 1.53 MPa under 2000 N axial load (Sfig. 9c). Furthermore, the maximum compressive stress of 3.87 MPa, in the lateral plateau was computed in the uncovered zone near the central area. A stiffer moment-rotation response has been computed under increased pure coupled rotation (adduction and internal rotations). For example, the internal and adduction moments reach their maximum value of 5.6 and 29.3 Nm at 12 deg and 6 deg rotation, respectively (Sfig. 9d). Our computed results under joint full extension posture have been corroborated by experimental measurements employing similar boundary conditions [33, 35, 38-41, 47-49].

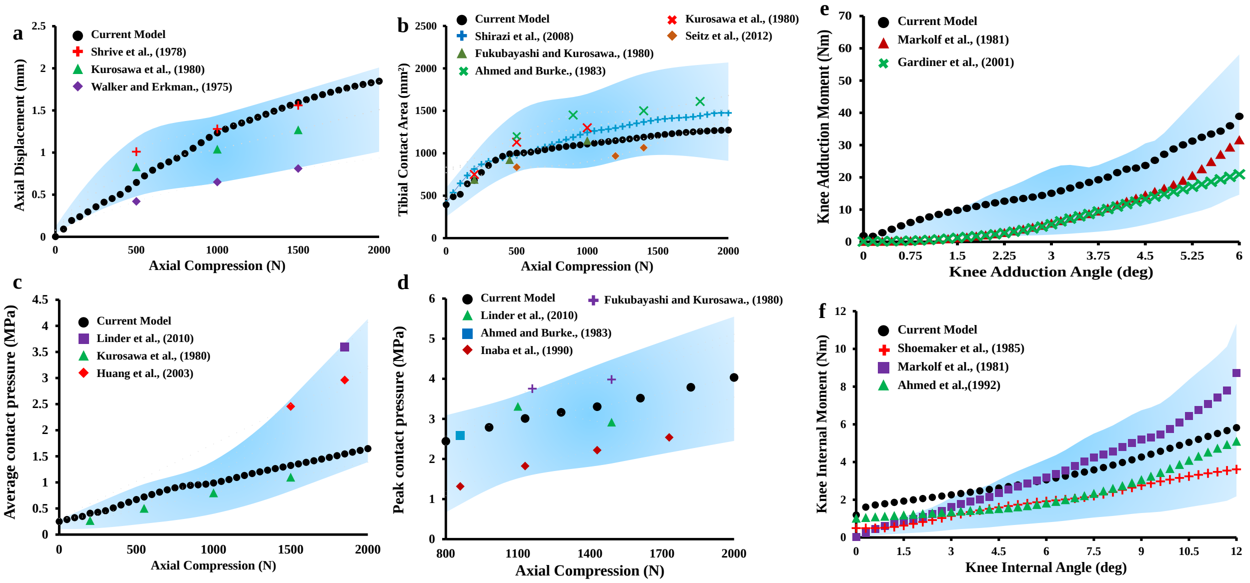

Supplementary Figure S(9): Tibial axial displacement (a), contact area (b), average and peak pressure (c, d) under axial compressive load up to 2000 N, tibial pure coupled moment-rotation (e,f), along with experimental measurements under almost the same boundary conditions. The highlighted area indicated the standard deviation of experimental measurements.

The tibial rolled on the femur posteriorly during passive knee flexion as the joint flexed to 90 degrees. The coupled joint laxities were directed laterally on the whole range of joint flexion

along the frontal axis while starting distally and switched at mid-range to proximally along the transversal axis (Sfig. 10). These joint translations were computed based on the position of the primary tibial node (tibial bone reference node), which may alter if another node has been selected. This may explain the slight computed differences with measured results under almost the same boundary conditions[37]. In addition, the coupled internal rotation at the tibia substantially increased at high flexion angles. However, a small adduction rotation was computed during knee flexion and was characterized by an almost linear behavior. Finally, the overall kinematics of the tibiofemoral joint was in good agreement with the earlier cadaveric study of Germain et al.,[37].

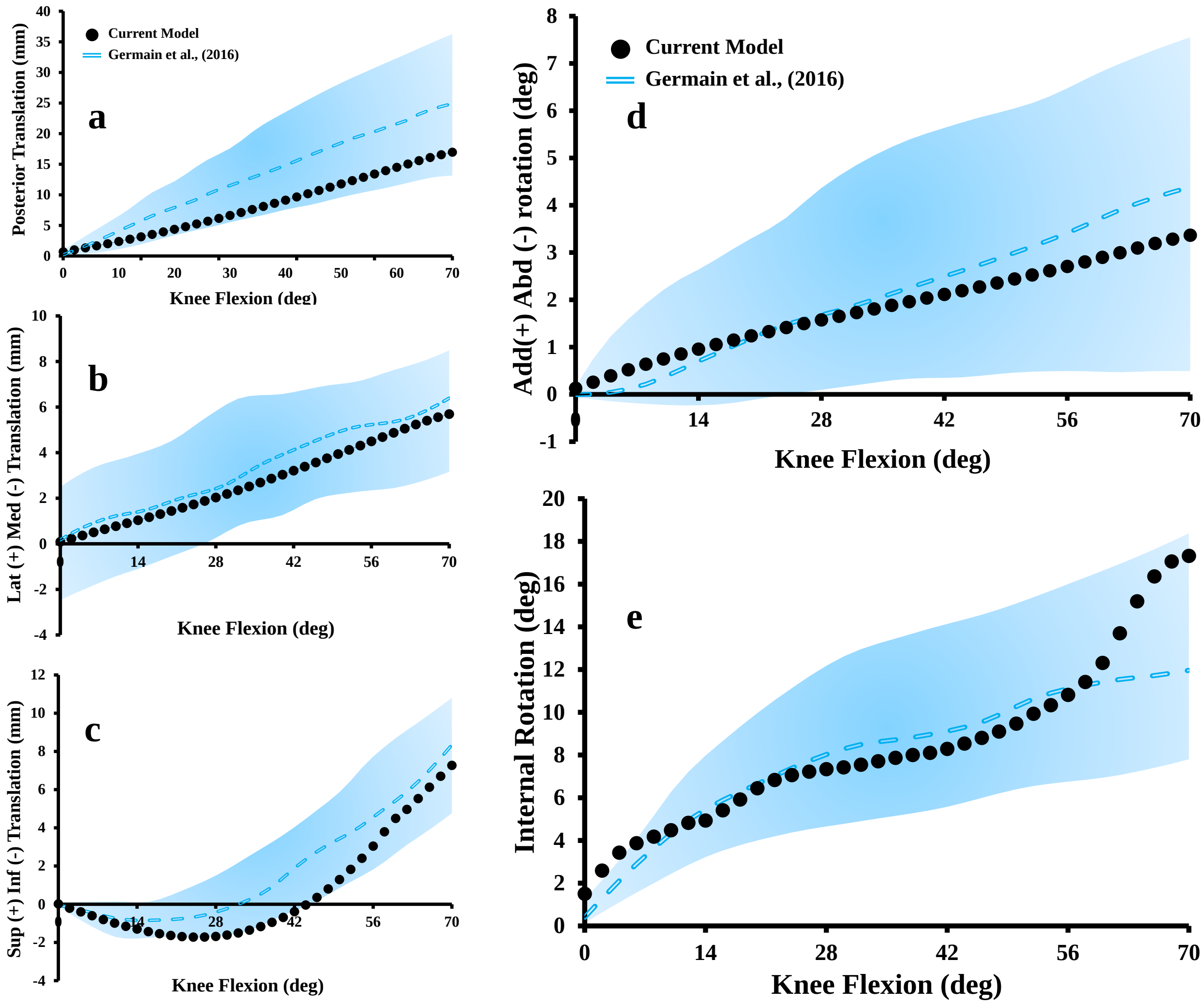

Supplementary Figure S(10): Kinematics of the tibiofemoral joint during passive knee flexion. The highlighted area indicated the standard deviation of experimental measurements. (a-c) tibial translation, (d-e) tibial coupled rotations.

For the forces in ligaments, The PCL force increased during knee flexion and reached its maximum value of 11 N at 90 degrees flexion. However, the ACL follows an opposite trend where a maximum of 77 N was computed at full extension and decreased after that on the rest of the range of joint flexion. The observed behavior confirms the primary role of the cruciate ligaments, in which the ACL is the primary component that resists anterior tibial translation at full extension. At the same time, the PCL remained slack and got active at higher angle flexion to resist associated posterior tibial translation. The following figure (Sfig. 11) compares computed cruciate ligament forces with computational prediction and experimental measurements under almost the same boundary conditions [43-45, 50].

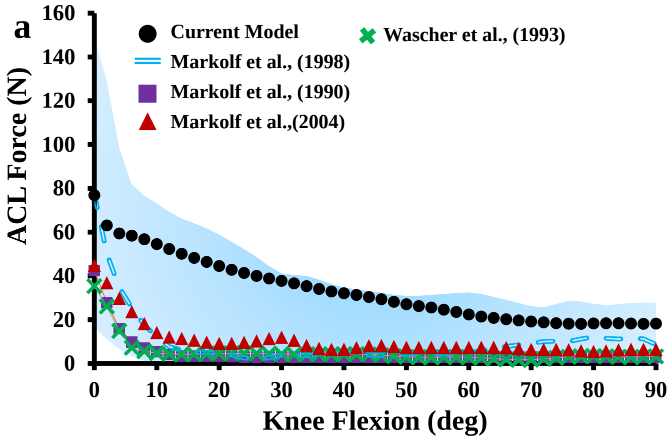

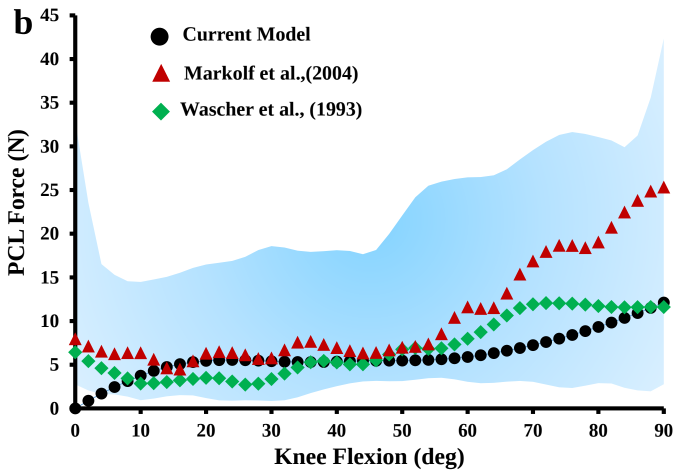

Supplementary Figure S(11): Comparison of our predicted and measured cruciate ligament forces during passive knee flexion. The highlighted area indicated the standard deviation of experimental measurements.

6. Muscles Optimization

A nonlinear optimization technique has been employed to evaluate the unknown muscle forces ($\{x\}$) at each instance of the stance phase of gait. This optimization procedure minimizes an objective function of the sum of cubed muscle stresses *($f(x_i)$)* (4) under the constraint that muscle forces remain positive between their passive forces and total maximum active forces (6). All muscles' passive and maximum active forces were driven from a scaled musculoskeletal model that matched the subject's dimension considered in our gait analyses [15]. The main constraint driving the evaluation of the muscle forces was the equilibrium equations (5).

$$f(x_i) = \sum_i^n \left(\frac{x_i}{PCSA_i}\right)^3 \qquad (4)$$

$$[R]\{x\} = \{M\} \qquad (5)$$

$$\{x_p\} \leq \{x\} \leq \{x_{max}\} \qquad (6)$$

With x_i , x_p , [R] , x_{max}, $PCSA_i$ are the force of muscle, a passive force of muscle, and lever arms matrix at different instances during the stance phase, maximum force, and physiological cross-sectional areas for muscle i, respectively [15]. $[M]$ are the (required) lower limb joint moments (hip, knee, and ankle) computed during the different instances of the stance phase (Sfig. 12).

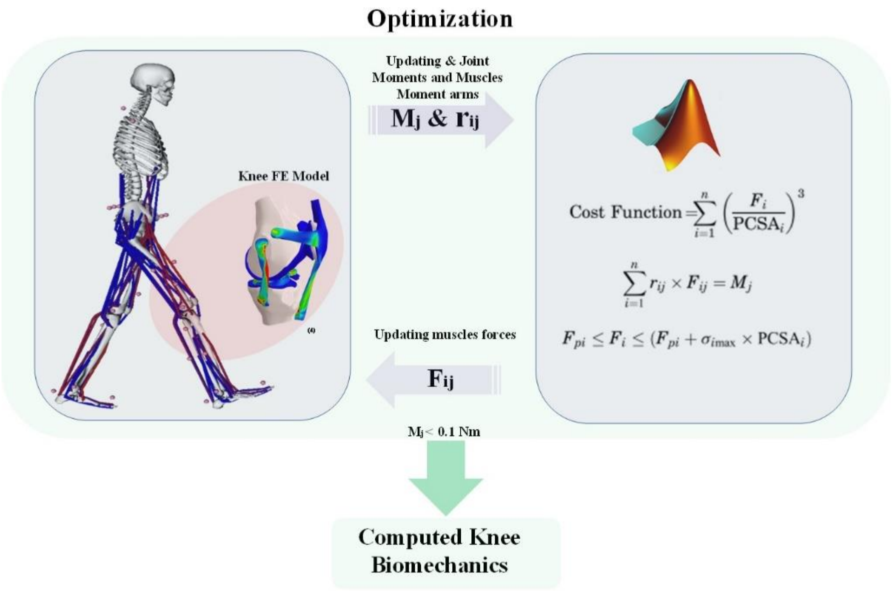

Supplementary Figure S(12): Procedure for estimating updated muscle forces during the stance phase of gait [52].

References:

1. Browning, R.C. and R. Kram, *Effects of obesity on the biomechanics of walking at different speeds.* Med Sci Sports Exerc, 2007. **39**(9): p. 1632-41.

2. DeVita, P. and T. Hortobagyi, *Obesity is not associated with increased knee joint torque and power during level walking.* J Biomech, 2003. **36**(9): p. 1355-62.

3. Freedman Silvernail, J., et al., *The influence of body mass index and velocity on knee biomechanics during walking.* Gait Posture, 2013. **37**(4): p. 575-9.

4. Li, J.-S., et al., *Six degree-of-freedom knee joint kinematics in obese individuals with knee pain during gait.* PloS one, 2017. **12**(3): p. e0174663.

5. Russell, E.M., *Lateral wedges and the biomechanical risk for knee osteoarthritis*. 2011, ProQuest Dissertations Publishing.

6. Faul, F., et al., *Statistical power analyses using G*Power 3.1: tests for correlation and regression analyses.* Behav Res Methods, 2009. **41**(4): p. 1149-60.

7. Faul, F., et al., *G*Power 3: a flexible statistical power analysis program for the social, behavioral, and biomedical sciences.* Behav Res Methods, 2007. **39**(2): p. 175-91.

8. Shultz, S.P., et al., *Body size and walking cadence affect lower extremity joint power in children's gait.* Gait Posture, 2010. **32**(2): p. 248-52.

9. Silva, F.R., et al., *Biomechanical alterations of gait on overweight subjects.* Research on Biomedical Engineering, 2018. **34**(4): p. 291-298.

10. Seidell, J.C. and K.M. Flegal, *Assessing obesity: classification and epidemiology.* British medical bulletin, 1997. **53**(2): p. 238-252.

11. Vince, A., A. Singhania, and M. Glasgow, *What knee X-rays do we need? A survey of orthopaedic surgeons in the United Kingdom.* The Knee, 2000. **7**(2): p. 101-104.

12. Whittle, M.W., *Clinical gait analysis: A review.* Human Movement Science, 1996. **15**(3): p. 369-387.

13. Wilken, J.M., et al., *Reliability and Minimal Detectible Change values for gait kinematics and kinetics in healthy adults.* Gait Posture, 2012. **35**(2): p. 301-7.

14. Kirtley, C., *Clinical gait analysis: theory and practice*. 2006: Elsevier Health Sciences.

15. Delp, S.L., et al., *OpenSim: open-source software to create and analyze dynamic simulations of movement.* IEEE Trans Biomed Eng, 2007. **54**(11): p. 1940-50.

16. Medved, V., et al., *Gait Analysis*, in *Measurement and Analysis of Human Locomotion*. 2022, Springer. p. 219-255.

17. Besier, T.F., et al., *Knee muscle forces during walking and running in patellofemoral pain patients and pain-free controls.* J Biomech, 2009. **42**(7): p. 898-905.

18. Erdemir, A., et al., *Model-based estimation of muscle forces exerted during movements.* Clin Biomech (Bristol, Avon), 2007. **22**(2): p. 131-54.

19. Erdemir, A., *Open knee: open source modeling & simulation to enable scientific discovery and clinical care in knee biomechanics.* The journal of knee surgery, 2016. **29**(2): p. 107.

20. Dhaher, Y.Y. and L.E. Kahn, *The effect of vastus medialis forces on patello-femoral contact: a model-based study.* J Biomech Eng, 2002. **124**(6): p. 758-67.

21. Donahue, T.L., et al., *A finite element model of the human knee joint for the study of tibio-femoral contact.* J Biomech Eng, 2002. **124**(3): p. 273-80.

22. Jahangir, S., et al., *Rapid X-Ray-Based 3-D Finite Element Modeling of Medial Knee Joint Cartilage Biomechanics During Walking.* Annals of Biomedical Engineering, 2022. **50**(6): p. 666-679.

23. Sajjadinia, S.S., M. Haghpanahi, and M. Razi, *Computational simulation of the multiphasic degeneration of the bone-cartilage unit during osteoarthritis via indentation and unconfined compression tests.* Proceedings of the Institution of Mechanical Engineers, Part H: Journal of Engineering in Medicine, 2019. **233**(9): p. 871-882.

24. Wilson, W., J.M. Huyghe, and C.C. van Donkelaar, *Depth-dependent compressive equilibrium properties of articular cartilage explained by its composition.* Biomech Model Mechanobiol, 2007. **6**(1-2): p. 43-53.

25. Tissakht, M. and A. Ahmed, *Tensile stress-strain characteristics of the human meniscal material.* Journal of biomechanics, 1995. **28**(4): p. 411-422.

26. Fithian, D.C., M.A. Kelly, and V.C. Mow, *Material properties and structure-function relationships in the menisci.* Clinical orthopaedics and related research, 1990(252): p. 19-31.

27. Proctor, C.S., et al., *Material properties of the normal medial bovine meniscus.* J Orthop Res, 1989. **7**(6): p. 771-82.

28. Adouni, M., T.R. Faisal, and Y.Y. Dhaher, *Computational frame of ligament in situ strain in a full knee model.* Computers in Biology and Medicine, 2020. **126**: p. 104012.

29. Staubli, H.U., et al., *Mechanical tensile properties of the quadriceps tendon and patellar ligament in young adults.* Am J Sports Med, 1999. **27**(1): p. 27-34.

30. Dhaher, T.H. Kwon, and M. Barry, *The effect of connective tissue material uncertainties on knee joint mechanics under isolated loading conditions.* J Biomech, 2010. **43**(16): p. 3118-25.

31. Limbert, G. and J. Middleton, *A transversely isotropic viscohyperelastic material - Application to the modeling of biological soft connective tissues.* International Journal of Solids and Structures, 2004. **41**(15): p. 4237-4260.

32. Aalbersberg, S., et al., *Orientation of tendons in vivo with active and passive knee muscles.* J Biomech, 2005. **38**(9): p. 1780-8.

33. Ahmed, A. and D. Burke, *In-Vitro of Measurement of Static Pressure Distribution in Synovial Joints—Part I: Tibial Surface of the Knee.* Journal of Biomechanical Engineering, 1983. **105**: p. 216.

34. Ahmed, A.M., et al., *Ligament tension pattern in the flexed knee in combined passive anterior translation and axial rotation.* J Orthop Res, 1992. **10**(6): p. 854-67.

35. Fukubayashi, T. and H. Kurosawa, *The contact area and pressure distribution pattern of the knee. A study of normal and osteoarthrotic knee joints.* Acta Orthop Scand, 1980. **51**(6): p. 871-9.

36. Gardiner, J.C., J.A. Weiss, and T.D. Rosenberg, *Strain in the human medial collateral ligament during valgus loading of the knee.* Clin Orthop Relat Res, 2001. **391**(391): p. 266-74.

37. Germain, F., et al., *Role of ligaments in the knee joint kinematic behavior: development and validation of a finite element model*, in *Computational biomechanics for medicine*. 2016, Springer. p. 15-26.

38. Huang, A., M.L. Hull, and S.M. Howell, *The level of compressive load affects conclusions from statistical analyses to determine whether a lateral meniscal autograft restores tibial contact pressure to normal: a study in human cadaveric knees.* J Orthop Res, 2003. **21**(3): p. 459-64.

39. Inaba, H.I., M.A. Arai, and W.W. Watanabe, *Influence of the varus-valgus instability on the contact of the femoro-tibial joint.* Proc Inst Mech Eng H, 1990. **204**(1): p. 61-4.

40. Kurosawa, H., T. Fukubayashi, and H. Nakajima, *Load-Bearing Mode of the Knee-Joint - Physical Behavior of the Knee-Joint with or without Menisci.* Clinical Orthopaedics and Related Research, 1980. **149**(149): p. 283-290.

41. Linder-Ganz, E., et al., *A novel quantitative approach for evaluating contact mechanics of meniscal replacements.* J Biomech Eng, 2010. **132**(2): p. 024501.

42. Markolf, K.L., et al., *The role of joint load in knee stability.* J Bone Joint Surg Am, 1981. **63**(4): p. 570-85.

43. Markolf, K.L., et al., *Direct measurement of resultant forces in the anterior cruciate ligament. An in vitro study performed with a new experimental technique.* J Bone Joint Surg Am, 1990. **72**(4): p. 557-67.

44. Markolf, K.L., et al., *Effects of applied quadriceps and hamstrings muscle loads on forces in the anterior and posterior cruciate ligaments.* Am J Sports Med, 2004. **32**(5): p. 1144-9.

45. Markolf, K.L., et al., *In situ calibration of miniature sensors implanted into the anterior cruciate ligament part I: Strain measurements.* Journal of Orthopaedic Research, 1998. **16**(4): p. 455-463.

46. Seitz, A., et al., *Forces acting on the anterior meniscotibial ligaments.* Knee Surg Sports Traumatol Arthrosc, 2012. **20**(8): p. 1488-95.

47. Shirazi, R., A. Shirazi-Adl, and M. Hurtig, *Role of cartilage collagen fibrils networks in knee joint biomechanics under compression.* J Biomech, 2008. **41**(16): p. 3340-8.

48. Shrive, N.G., J.J. O'Connor, and J.W. Goodfellow, *Load-bearing in the knee joint.* Clin Orthop Relat Res, 1978. **131**(131): p. 279-87.

49. Walker, P.S. and M.J. Erkman, *The role of the menisci in force transmission across the knee.* Clin Orthop Relat Res, 1975. **109**(109): p. 184-92.

50. Wascher, D.C., et al., *Direct in vitro measurement of forces in the cruciate ligaments. Part I: The effect of multiplane loading in the intact knee.* J Bone Joint Surg Am, 1993. **75**(3): p. 377-86.

51. Shoemaker, S.C. and K.L. Markolf, *Effects of joint load on the stiffness and laxity of ligament-deficient knees. An in vitro study of the anterior cruciate and medial collateral ligaments.* J Bone Joint Surg Am, 1985. **67**(1): p. 136-46.

52. Adouni, M., *Analyse biomécanique de l'articulation de genou durant la bipédie humaine*. 2014, École polytechnique de Montréal.
